# The Impact of Racism on Health: A Health Equity Training on Structural Racism for Military Residents and Fellows

**DOI:** 10.15766/mep_2374-8265.11443

**Published:** 2024-09-12

**Authors:** Veronica Wright, Emily Stepp, Brittany Flemming, Courtney Kandler, Tirzah Wait, Loxley Godshall-Bennett, Rachel Robbins

**Affiliations:** 1 Fourth-Year Resident, Department of Medicine & Department of Psychiatry, Walter Reed National Military Medical Center; 2 Second-Year Resident, Department of Medicine & Department of Psychiatry, Walter Reed National Military Medical Center; 3 Fellow, Department of Adolescent Medicine, San Antonio Uniformed Services Health Education Consortium; 4 Fellow, Department of Child and Adolescent Psychiatry, Walter Reed National Military Medical Center; 5 Fourth-Year Resident, Department of Radiology, Walter Reed National Military Medical Center; 6 Second-Year Resident, Department of Psychiatry, Walter Reed National Military Medical Center; 7 Associate Professor, Department of Rheumatology, Walter Reed National Military Medical Center

**Keywords:** Diversity, Equity, Inclusion, Case-Based Learning, Antiracism, Bias, Health Disparities

## Abstract

**Introduction:**

Recognizing the need for more opportunities to learn about health equity within military graduate medical education (GME), we developed a resident-led curriculum to introduce these concepts from a military cultural competency lens. The Impact of Racism on Health module focuses on structural racism and health disparities.

**Methods:**

This 60-minute module was presented to ear, nose, and throat (ENT) and pediatrics residents and fellows. It includes a case presentation of an adolescent with an asthma exacerbation, a large-group discussion about social determinants of health and structural racism, and a small-group discussion/debrief conceptualizing the case.

**Results:**

Thirty pediatrics residents and 15 ENT residents participated in this activity with a 46% and 60% pretest response rate, respectively. A two-sample Mann–Whitney *U* test showed statistically significant improvement (*p* = .005) in knowledge related to structural racism between the pretest (*M* = 0.5, *SD* = 0.3) and posttest (*M* = 0.7, *SD* = 0.1) knowledge assessments with a small effect size (*r* = 0.4; Z = 2.8).

**Discussion:**

We demonstrated that interactive teaching methods can be used to educate military GME trainees on the impact of structural racism on health outcomes for military health care beneficiaries. Understanding the role of structural racism in the context of military health care using curricula that highlight military-specific health disparities is essential to understanding the role of the military physician in systemically addressing health disparities.

## Educational Objectives

By the end of this activity, learners will be able to:

1. Define and describe social determinants of health.
2. Generate examples of modifiable and nonmodifiable risk factors that impact the health outcomes of military health care beneficiaries.
3. Identify social determinants of health as possible barriers to addressing modifiable risk factors.
4. Recognize redlining as an example of structural racism.

## Introduction

The Accreditation Council for Graduate Medical Education (ACGME) has acknowledged the impact of diversity, equity, and inclusion (DEI) on health outcomes and charged the graduate medical education (GME) community with prioritizing diversity, equity, and inclusion at all levels.^1^ Many of the resources meant to assist with DEI teaching exist in the form of articles related to health care disparities, or online learning modules intended for independent, self-directed professional growth (e.g., the ACGME Equity Matters site or AMA's Center for Health Equity Ed Hub).^2,3^ These both have advantages in their ease of access and the diverse DEI professionals teaching health equity concepts, but few resources have published curricula.

The *MedEdPORTAL* DEI Collection includes several published curricula, but many of these are specialty- or population-specific,^4^ and none address health equity in the context of the military health care system (MHS). Additionally, military GME programs, especially smaller programs, often lack guidance for teaching trainees DEI concepts in a context that is applicable to their patient population, and lack the personnel who are equipped and comfortable to develop and teach such curricula.

To address this need, we designed a curriculum to assist military GME programs with initiating introductory DEI conversations through the lens of military cultural competency using the Kern 6-step approach.^5^ Our primary objective was for the curriculum to align with ACGME's equity-focused common program requirements (i.e., competence in cultural humility, respect and responsiveness to diverse patient populations, and incorporating considerations of equity in patient and population-based care)^6^ and increase DEI knowledge across multiple GME programs within our institution.

This module, The Impact of Racism on Health, is one of five stand-alone modules in our curriculum. The other modules within this curriculum include Implicit Bias, Institutional Prejudices, Cultural Diversity, and Inclusive Culture. At the time of developing the module and preparing this manuscript, no published curricula focused on the impact of structural racism on military beneficiary health outcomes, so this module is unique and additive to existing DEI curricula. Moreover, documented racial disparities in the MHS in areas such as diabetes readmissions, opioid usage, and minimally invasive women's health^7^ suggest that factors beyond just the presence of health insurance or employment also influence disparities. Therefore, introducing curricula highlighting disparities from the MHS using a structural racism approach is crucial for educating military physicians to address racial health disparities in this unique patient population.

## Methods

We (a group of residents, fellows, and two faculty advisors) developed a five-part DEI curriculum as members of the DEI Committee at Walter Reed National Military Medical Center. The project was granted IRB exempt status at our institution. Curriculum developers were from different specialties (internal medicine, pediatrics, psychiatry, physical medicine & rehabilitation, radiation oncology, and adult hematology/oncology) and each had various experiences in public health, clinical care, and DEI education. The Impact of Racism on Health module described in this publication is one part of this curriculum developed using Kern's 6-Step approach.^5^

### Step 1: Problem Identification and Needs Assessment

Development of this module was a dynamic process. Our DEI Committee recognized a need for more opportunities for GME trainees to have formal DEI discussions. Before implementing our curriculum, many of our programs had yet to dedicate a didactic session to addressing these topics. In discussing our collective past experiences and the known scarcity of curricula focused on the application of DEI concepts in military health care, we determined that the ideal approach would be a curriculum that could be a primer on DEI concepts through a lens of military cultural competency.

### Step 2: Targeted Needs Assessment

We conducted informal interviews of a subset of DEI committee representatives on their exposure to structural racism teaching in the past to identify the current practices of our GME programs and perceived best practices. Our informal interview of committee members’ prior experiences with anti-racism education revealed a preference for small group discussion, reflection, and case-based learning when being taught this material.

We also conducted a literature search of structural racism curricula. From this work, we identified structural racism as a gap in current DEI education within most of our GME programs and were able to identify teaching approaches that were successful in other anti-racism curricula.^8–11^

### Step 3: Goals and Objectives

Based on our needs assessment we compiled a list of DEI concepts to focus on for this curriculum and literature review, which included structural racism and social determinants of health. Thereafter, we decided upon specific learning objectives for this module based on what we perceived as core topics and skills related to structural racism.

### Step 4: Educational Strategies

We selected a combination of educational strategies aligned with our learning objectives and literature review: traditional lecture for introducing concepts, large-group discussion to explore understandings of concepts, and small-group discussion to allow for reflection and case-based learning. We included a case representative of a known racial health disparity in the MHS from the literature: pediatric asthma outcomes. This was included to encourage the application of structural racism and social determinants of health lenses to a clinical scenario familiar to our target audience as consequence of factors (e.g., absence of health insurance or job opportunities) that lead to health disparities in many other communities which are not typically faced by ethnic/racial minorities in our patient population.

### Step 5: Implementation

The Impact of Racism on Health module (Appendix A) was designed to be approximately 1-hour, including 30 minutes of didactic material with large-group discussion prompts throughout the presentation followed by 20 minutes of small-group work.

The session began in a large classroom with seats arranged in circles of four to six chairs for small groups that were not preassigned. Learners began by completing a pretest (Appendix B) in Google Forms via a QR code within the presentation. Next, the facilitator presented the didactic portion of the module for approximately 25 minutes, which provided the core content for each learning objective. The last section of the module was used for small-group case-based learning where learners discussed the case for 15 minutes. The small-group activity was followed by a 10-minute debrief where each small-group provided one or two examples of structures in the case that might be impacted by racism and how this knowledge might change the way they counsel the patient and family.

We presented the module as part of required didactic sessions for all residents within both programs. We designed it to be generalizable to GME trainees of other specialties as well.

Two residents who helped develop the module facilitated the presentations. Neither facilitator had significant experience with anti-racism teaching prior to facilitating so prepared for this presentation by reviewing the facilitator guide (Appendix C) and doing practice runs with codevelopers. Feedback from the other developers was used to revise the facilitator guide to a more user-friendly version that future facilitators could use to adequately prepare.

Between the first and second implementation of this module, we included additional literature about pediatric asthma disparities in the MHS due to the first facilitator's observation that many participants were not aware that this data existed and were questioning the applicability of the module's content and case example.

### Step 6: Evaluation and Feedback

We developed pre- and posttest knowledge assessment (Appendix B) related to the learning objectives to assess this module. The development was not based on anything previously used or published. No pilot testing was completed.

We collated assessment data for our analysis. Given concerns about the nonnormal continuous data from the collected assessments and an inability to pair the data, the data informatics team at Walter Reed National Military Medical Center's partnering institution, Uniformed Services University of the Health Sciences, was consulted for analytic assistance. We used a two-sample Mann–Whitney *U* test for data analysis at their recommendation.

## Results

This module was presented to both the pediatric and ENT residency programs at Walter Reed National Military Medical Center in 2022. Thirty residents in the pediatrics residency program and 15 residents in the ENT program participated with a pretest response rate of 46% and 60%, respectively. The pretest was completed by 24 residents (14 pediatrics, 9 ENT, 1 anesthesiology).

The posttest was completed by 20 residents (13 pediatrics, 7 ENT). The posttest had a significantly higher mean score (*M* = 0.7, *SD* = 0.1) than the pretest (*M* = 0.5, *SD* = 0.3; *p* = .005; Table). The difference between pretest and posttest means was calculated to have a small effect size (Z = 2.8; *r* = 0.4).

**Table.**
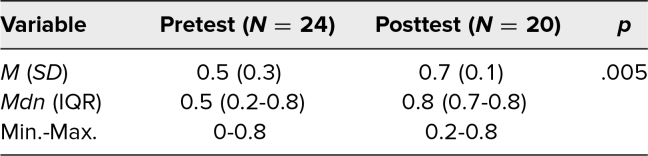
Participant Change in Knowledge Scores

## Discussion

We developed our GME DEI curriculum to introduce health equity concepts to military GME programs across different specialties. This module, The Impact of Racism on Health, provides an opportunity to apply knowledge of structural racism and social determinants of health to a case from a unique patient population (i.e., military health care beneficiaries). This is incredibly important given the subtlety of drivers for health disparities in patient populations that might not be impacted by more easily recognizable consequences of structural racism like absence of health insurance or stable employment. Emphasizing health disparities research like the one referenced in our module about disparities in military dependent's sponsor rank correlating with disparities in asthma prevalence and treatment for Black and Hispanic children receiving care in the MHS^12^ can prime learners to consider other structures that might be impacted by systemic racism like education funding policies, military policies that might lead to financial insecurity despite stable income, and housing policies. This approach can also be generalized to non-military settings with patient populations that have similar social determinants of health (i.e., health insurance and employment).

The development of this module presented both successes and challenges. The participation of residents and fellows from different specialties and a broad range of experiences was a strength in the development process. Neither of the facilitators had significant experience with anti-racism teaching and the feedback from the group both helped the delivery of this module and refinement of the facilitator guide. A significant challenge of using the Kern model to develop this module was the time-intensive approach it requires for both the preparation and implementation stages. Of note, we ultimately decided not to do a formal needs assessment of the residents in our GME DEI Committee since we knew that this group of individuals had skewed DEI experiences compared to our target audience and didn't have the time or resources to formally survey residents across our GME community which is a weakness of our study that could be addressed in future iterations.

While our data supports our objectives were achieved, there are some limitations and areas for improvement. First, we had a low survey response rate and relied on aggregated data rather than individual scores. To enhance study validity in the future, we aim to include anonymous personal identifiers to pair data. Additionally, we opted not to include demographic questions to keep assessments brief which limited our ability to analyze by race, age, and prior DEI involvement which could be helpful for subgroup analyses. Regarding content generalizability, the case used in this module was presented only to pediatrics and ENT residency programs, and it is unclear if it would have the same impact with other medical specialties. Additionally, while the module's concepts may apply to non-military GME programs, it is unknown if non-military participants would grasp nuances unique to the military (e.g., rank structure and housing norms) essential for understanding the case. To address both concerns, including free response questions in the assessment to elicit feedback on the activity could provide valuable data. Lastly, this was a single intervention with unknown long-term impact, so follow-up to determine if learning is sustained would be beneficial.

In conclusion, we determined that our module was effective in increasing military residents’ knowledge of the role of structural racism on health outcomes. We intend to continue improving our module based on the data collected and the limitations described above, with a long-term goal of disseminating this module to training facilities throughout the MHS. We believe that by implementing evidence-based, data-driven DEI training in military GME training programs, we can improve the perception of the importance of this topic and encourage trainees to adopt this knowledge in their medical practice.

## Appendices


Impact of Racism on Health Module.pptxPre- & Posttest.docxFacilitator Guide.docx
*All appendices are peer reviewed as integral parts of the Original Publication.*
