# Supplementary material for: The Impact of Racism on Health: A Health Equity Training on Structural Racism for Military Residents and Fellows
Source: MedEdPORTAL. 2024 Sep 12;20:11443. doi: 10.15766/mep_2374-8265.11443 (PMC11390879; doi:10.15766/mep_2374-8265.11443)
Supplement: Supplementary file 1 — Impact of Racism on Health Module.pptxPre- & Posttest.docxFacilitator Guide.docx [file mep_2374-8265.11443-s001.zip › A. Impact of Racism on Health Module.pptx]

## Slide 1
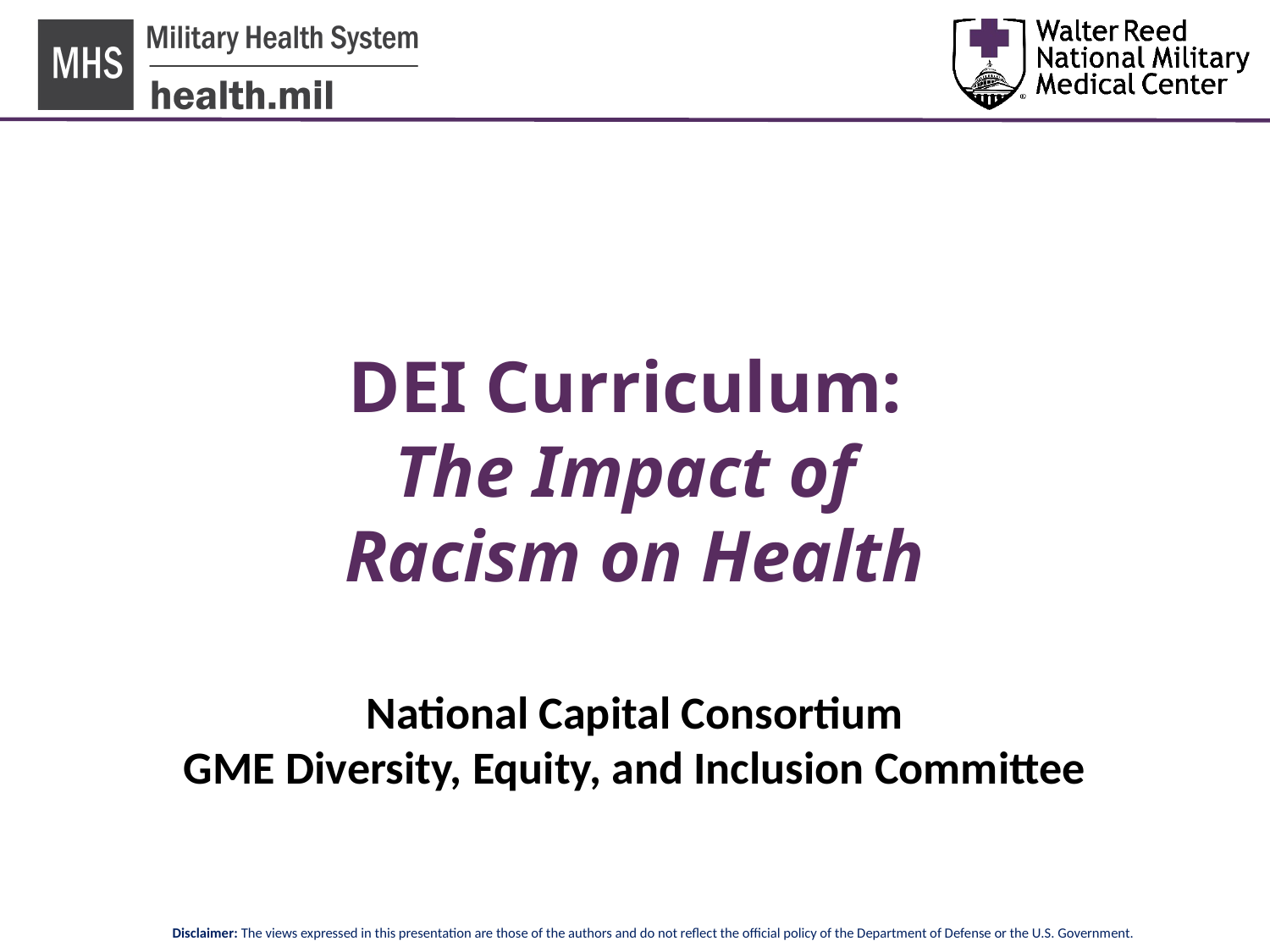

# DEI Curriculum: The Impact of Racism on Health
National Capital Consortium
GME Diversity, Equity, and Inclusion Committee

## Slide 2
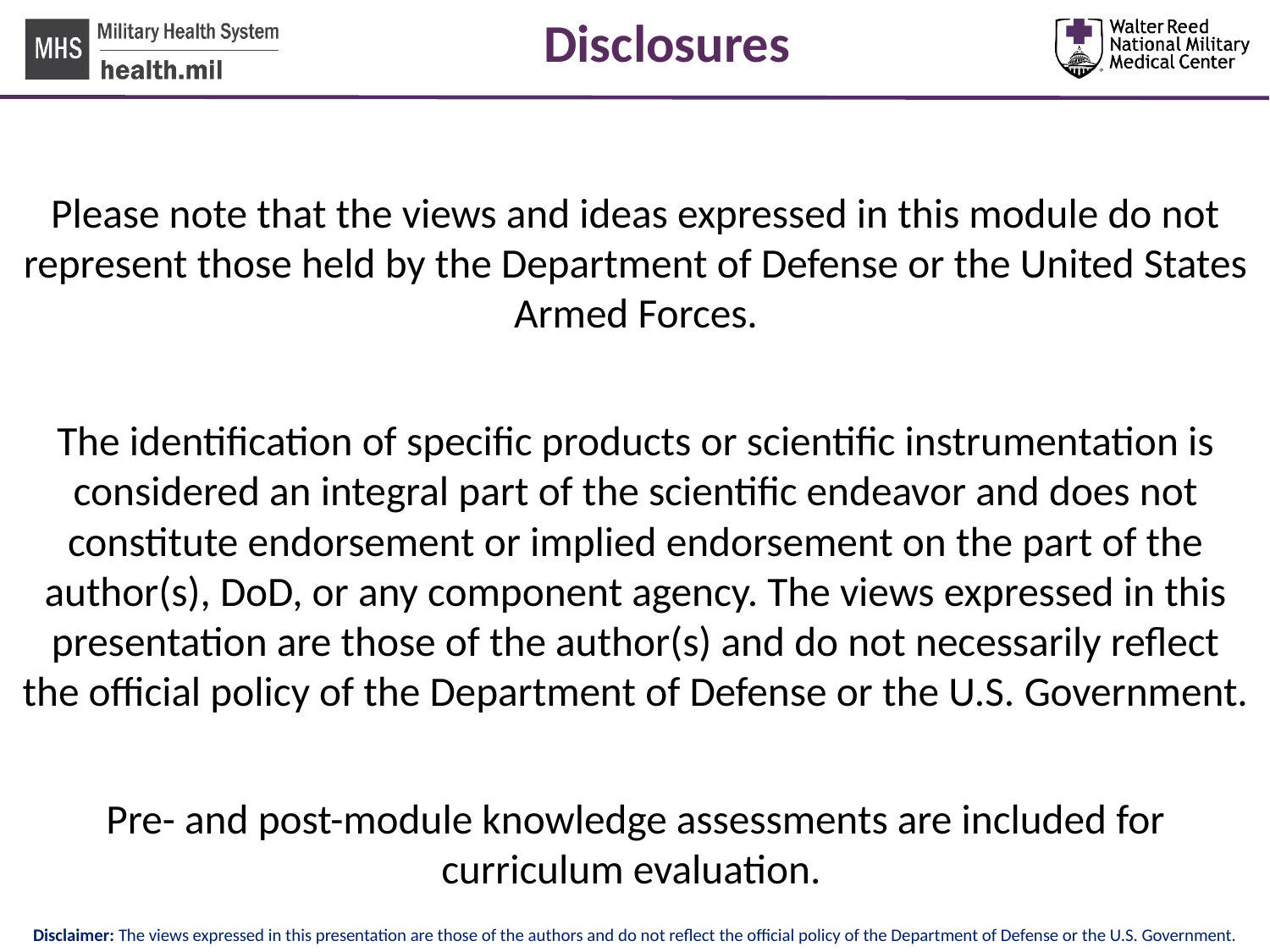

# Disclosures
Please note that the views and ideas expressed in this module do not represent those held by the Department of Defense or the United States Armed Forces.
The identification of specific products or scientific instrumentation is considered an integral part of the scientific endeavor and does not constitute endorsement or implied endorsement on the part of the author(s), DoD, or any component agency. The views expressed in this presentation are those of the author(s) and do not necessarily reflect the official policy of the Department of Defense or the U.S. Government.
Pre- and post-module knowledge assessments are included for curriculum evaluation.

## Slide 3
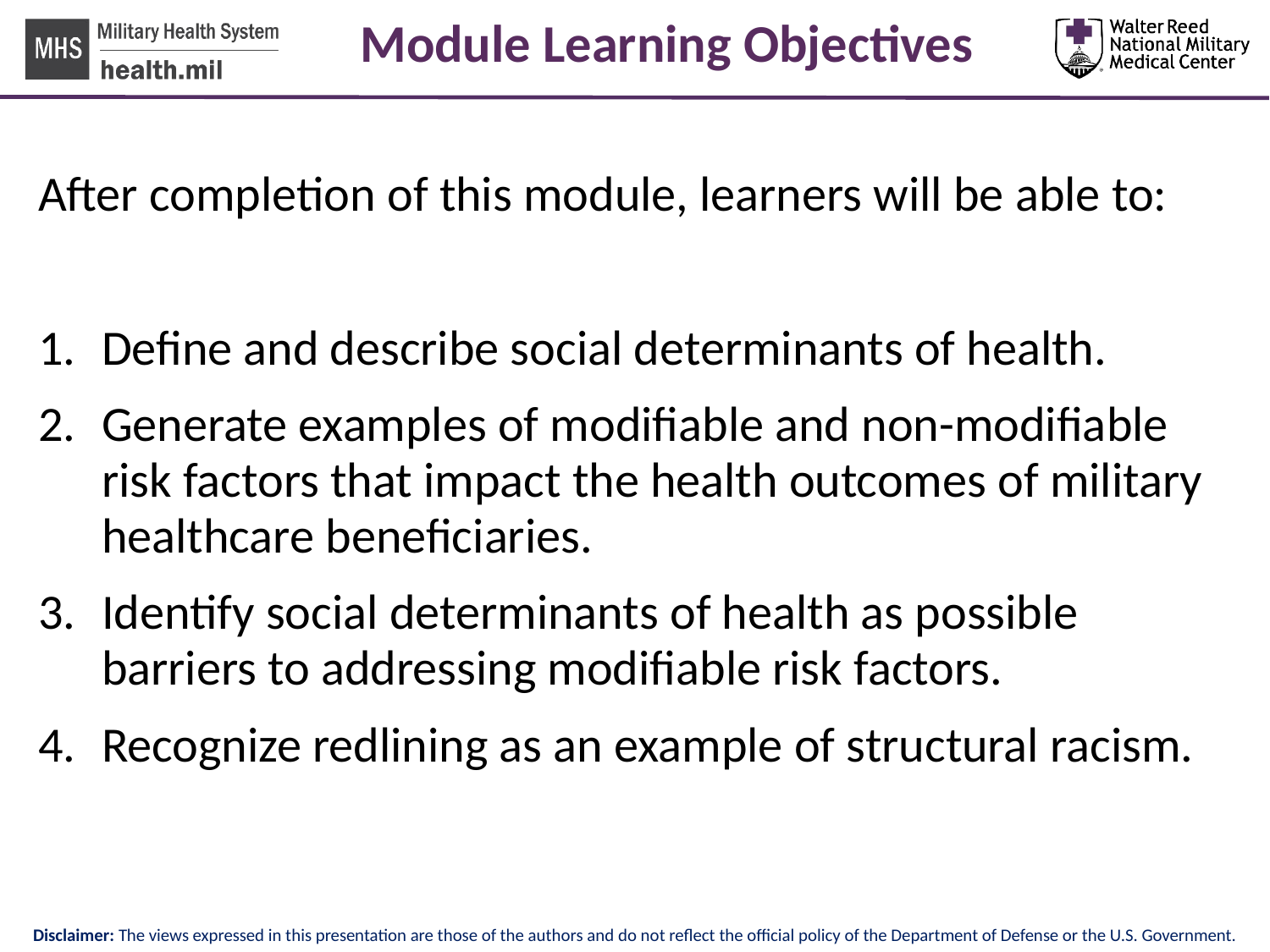

# Module Learning Objectives
After completion of this module, learners will be able to:
Define and describe social determinants of health.
Generate examples of modifiable and non-modifiable risk factors that impact the health outcomes of military healthcare beneficiaries.
Identify social determinants of health as possible barriers to addressing modifiable risk factors.
Recognize redlining as an example of structural racism.

## Slide 4
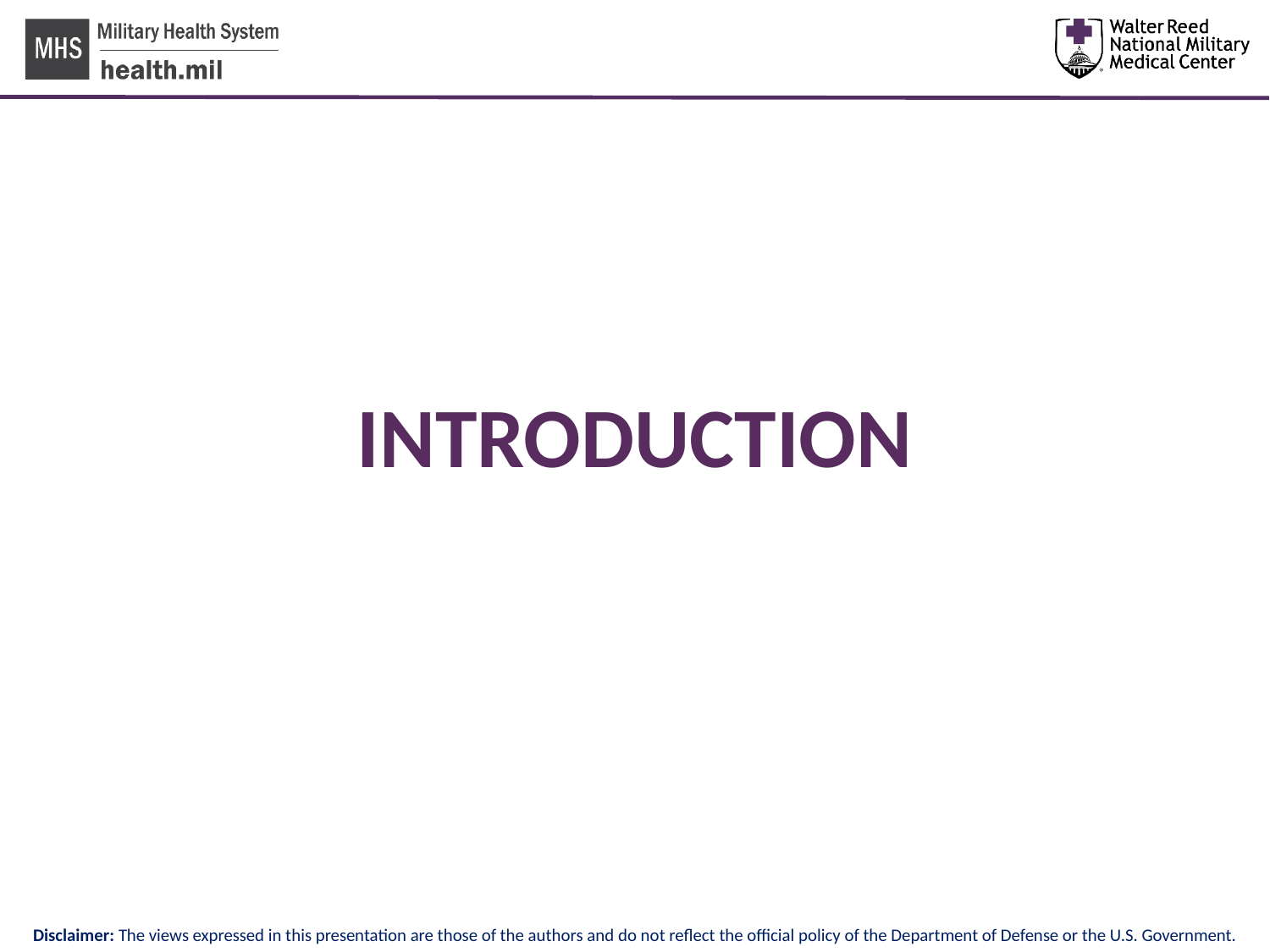

#
INTRODUCTION

## Slide 5
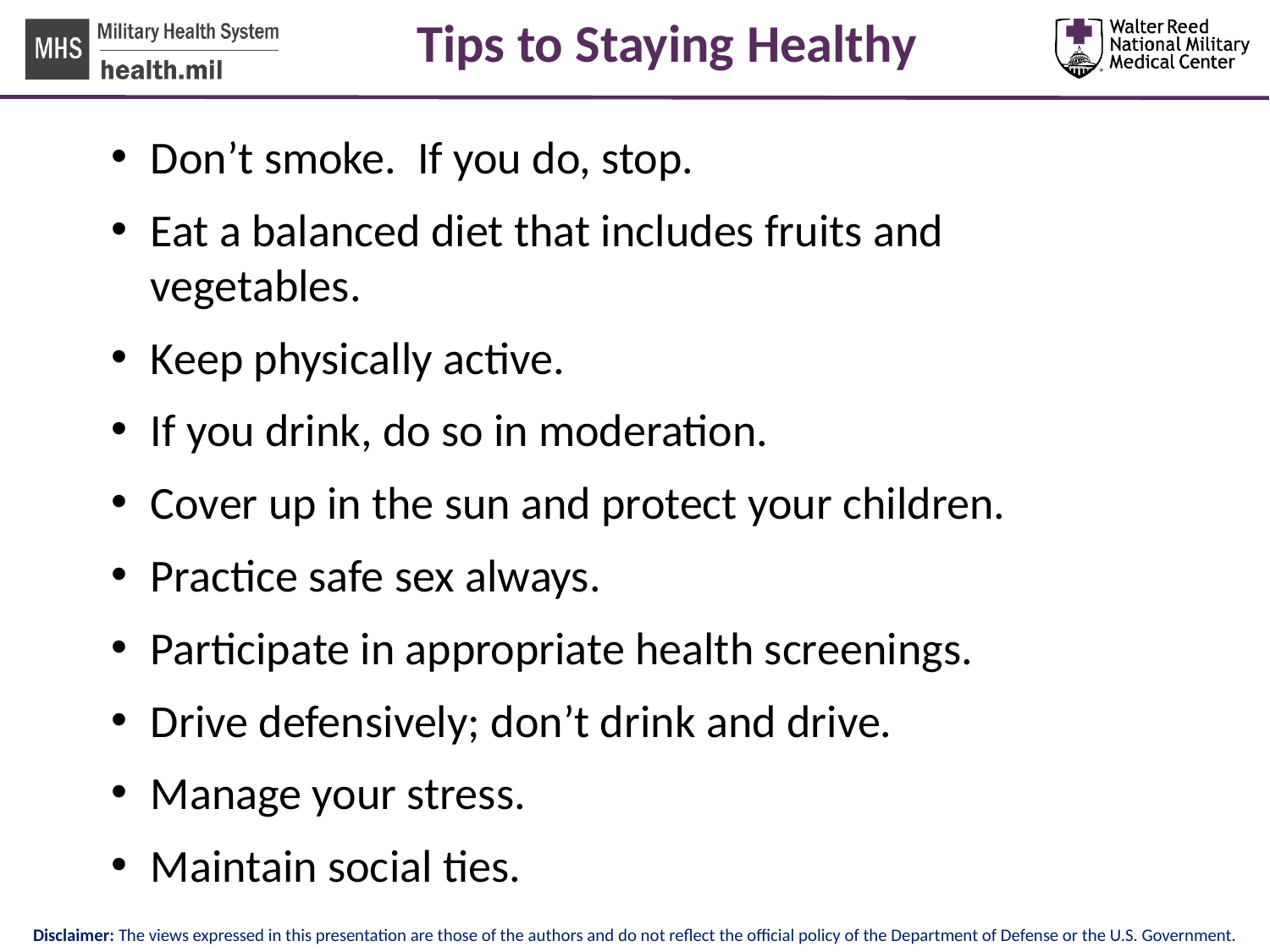

# Tips to Staying Healthy
Don’t smoke. If you do, stop.
Eat a balanced diet that includes fruits and vegetables.
Keep physically active.
If you drink, do so in moderation.
Cover up in the sun and protect your children.
Practice safe sex always.
Participate in appropriate health screenings.
Drive defensively; don’t drink and drive.
Manage your stress.
Maintain social ties.

## Slide 6
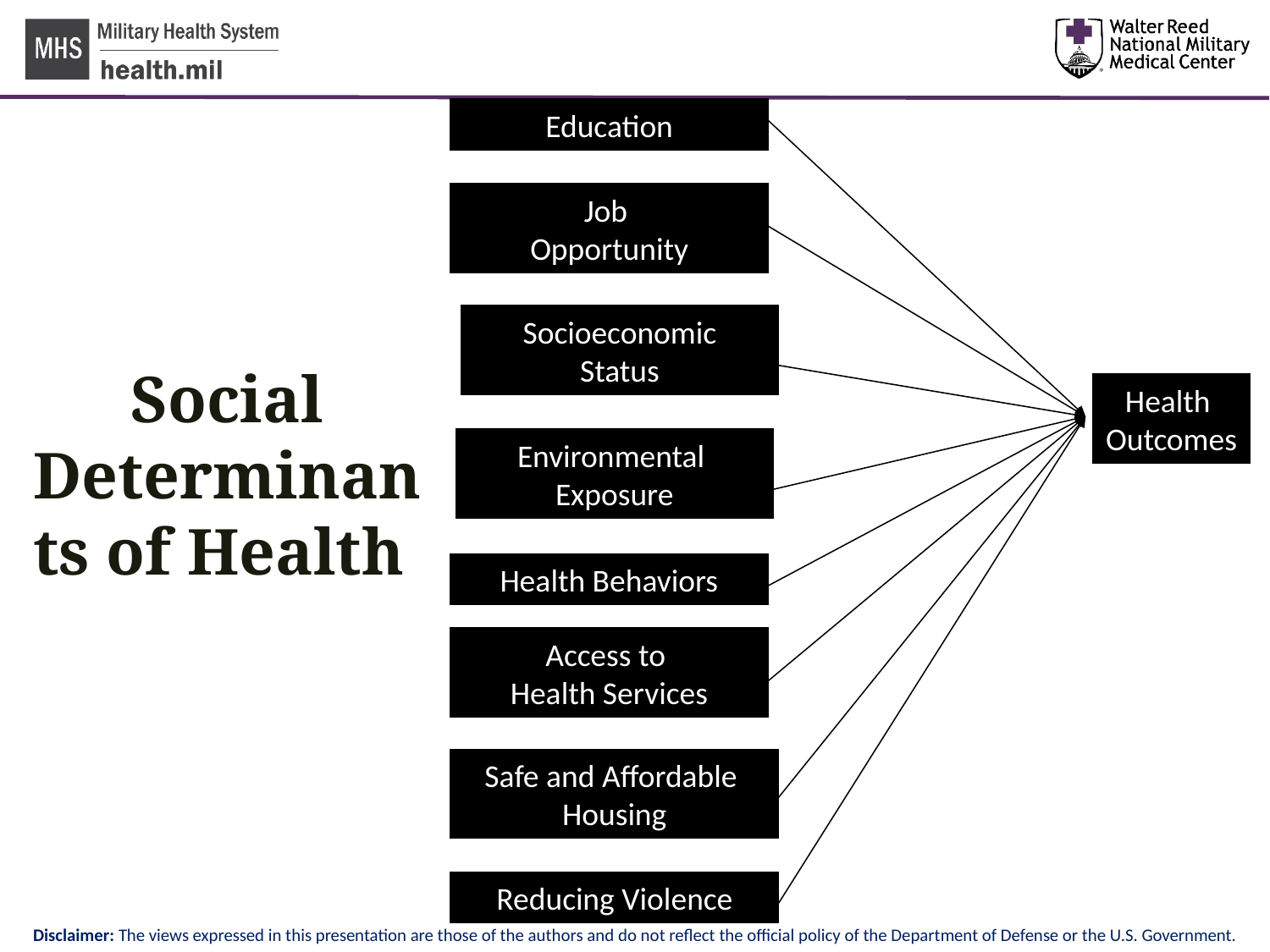

Education
Job
Opportunity
Socioeconomic
Status
Social Determinants of Health
Health
Outcomes
Environmental
Exposure
Health Behaviors
Access to
Health Services
Safe and Affordable
Housing
Reducing Violence

## Slide 7
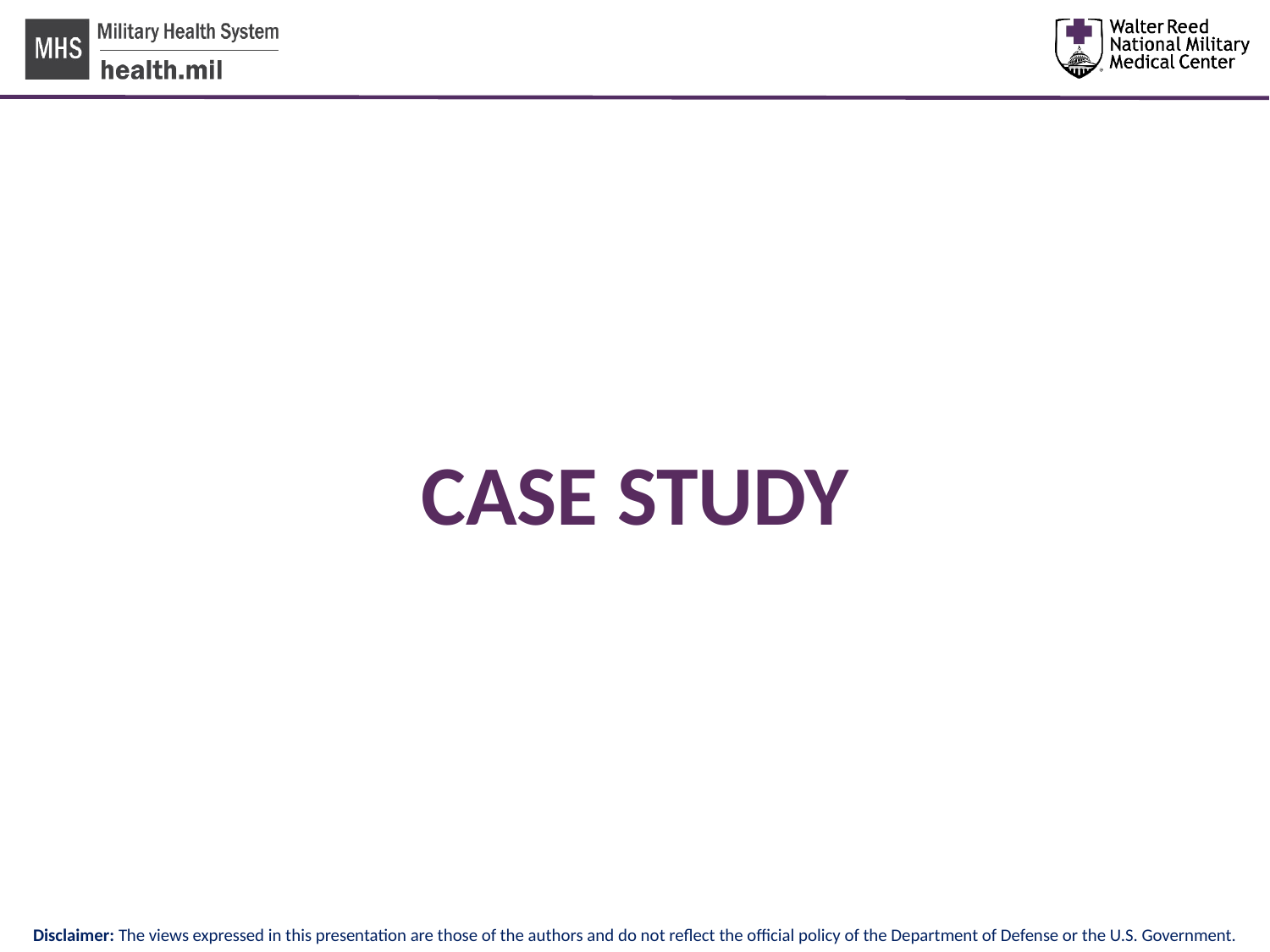

#
CASE STUDY

## Slide 8
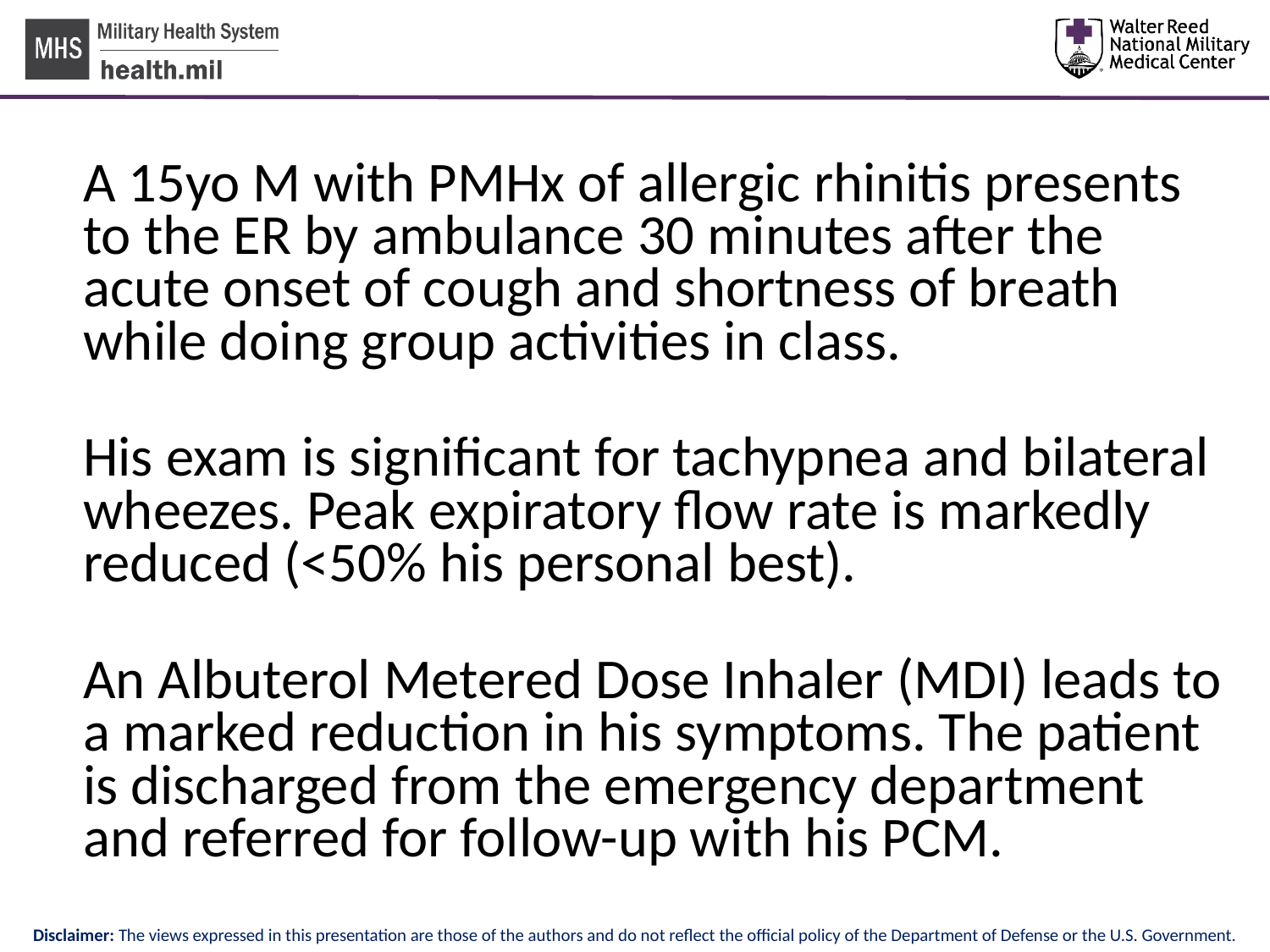

#
A 15yo M with PMHx of allergic rhinitis presents to the ER by ambulance 30 minutes after the acute onset of cough and shortness of breath while doing group activities in class.
His exam is significant for tachypnea and bilateral wheezes. Peak expiratory flow rate is markedly reduced (<50% his personal best).
An Albuterol Metered Dose Inhaler (MDI) leads to a marked reduction in his symptoms. The patient is discharged from the emergency department and referred for follow-up with his PCM.

## Slide 9
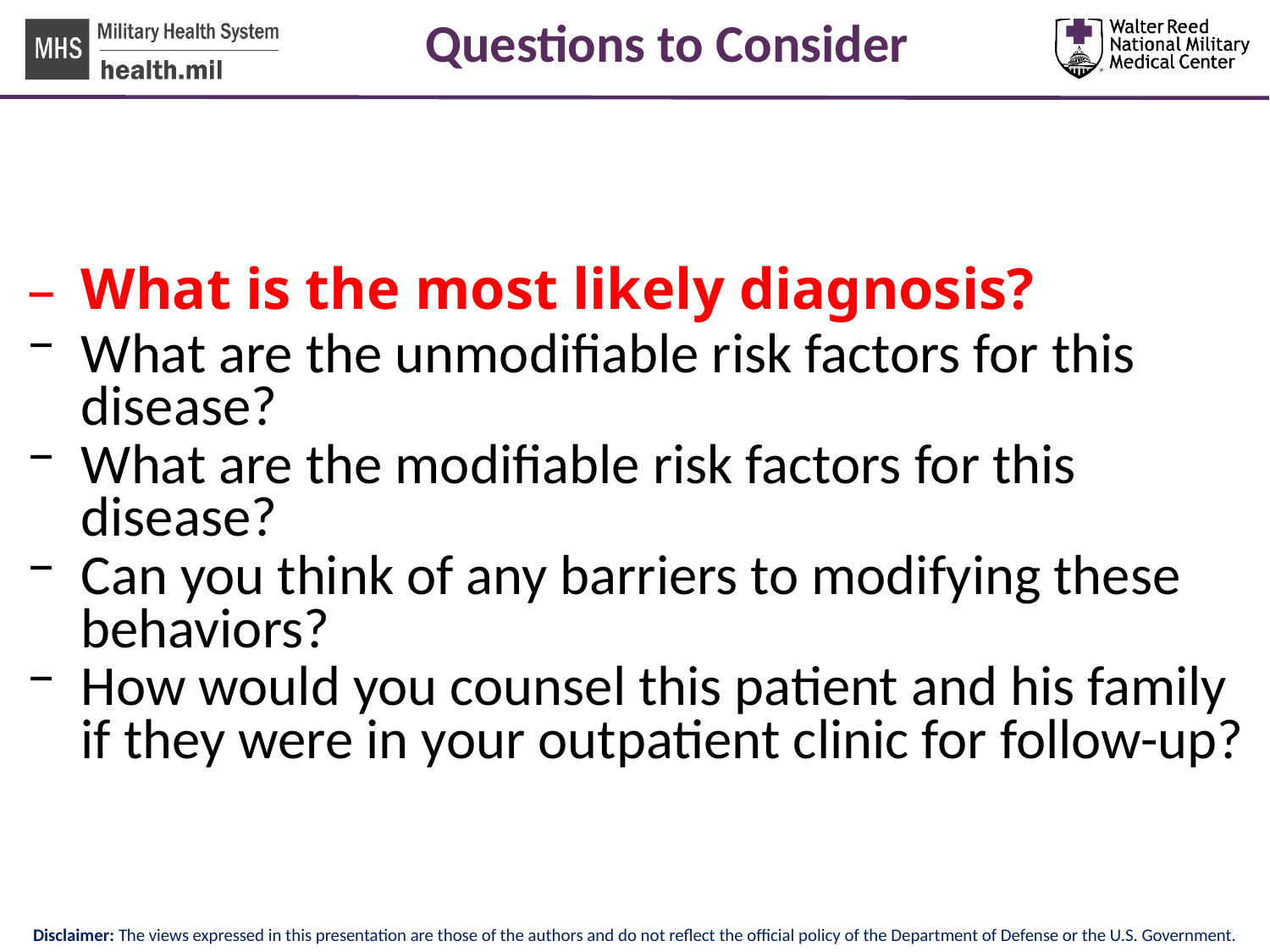

# Questions to Consider
What is the most likely diagnosis?
What are the unmodifiable risk factors for this disease?
What are the modifiable risk factors for this disease?
Can you think of any barriers to modifying these behaviors?
How would you counsel this patient and his family if they were in your outpatient clinic for follow-up?

## Slide 10
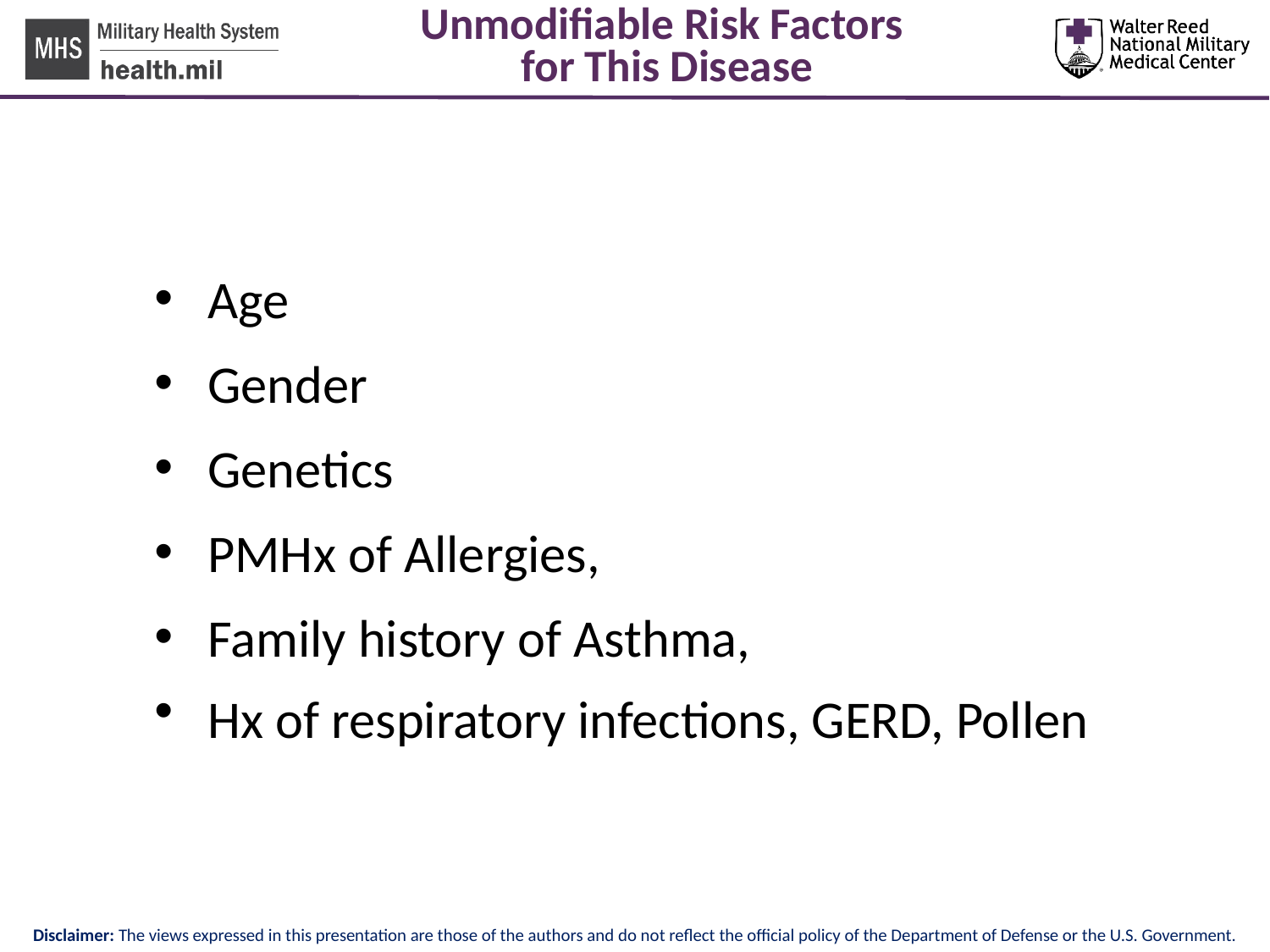

# Unmodifiable Risk Factors for This Disease
Age
Gender
Genetics
PMHx of Allergies,
Family history of Asthma,
Hx of respiratory infections, GERD, Pollen

## Slide 11
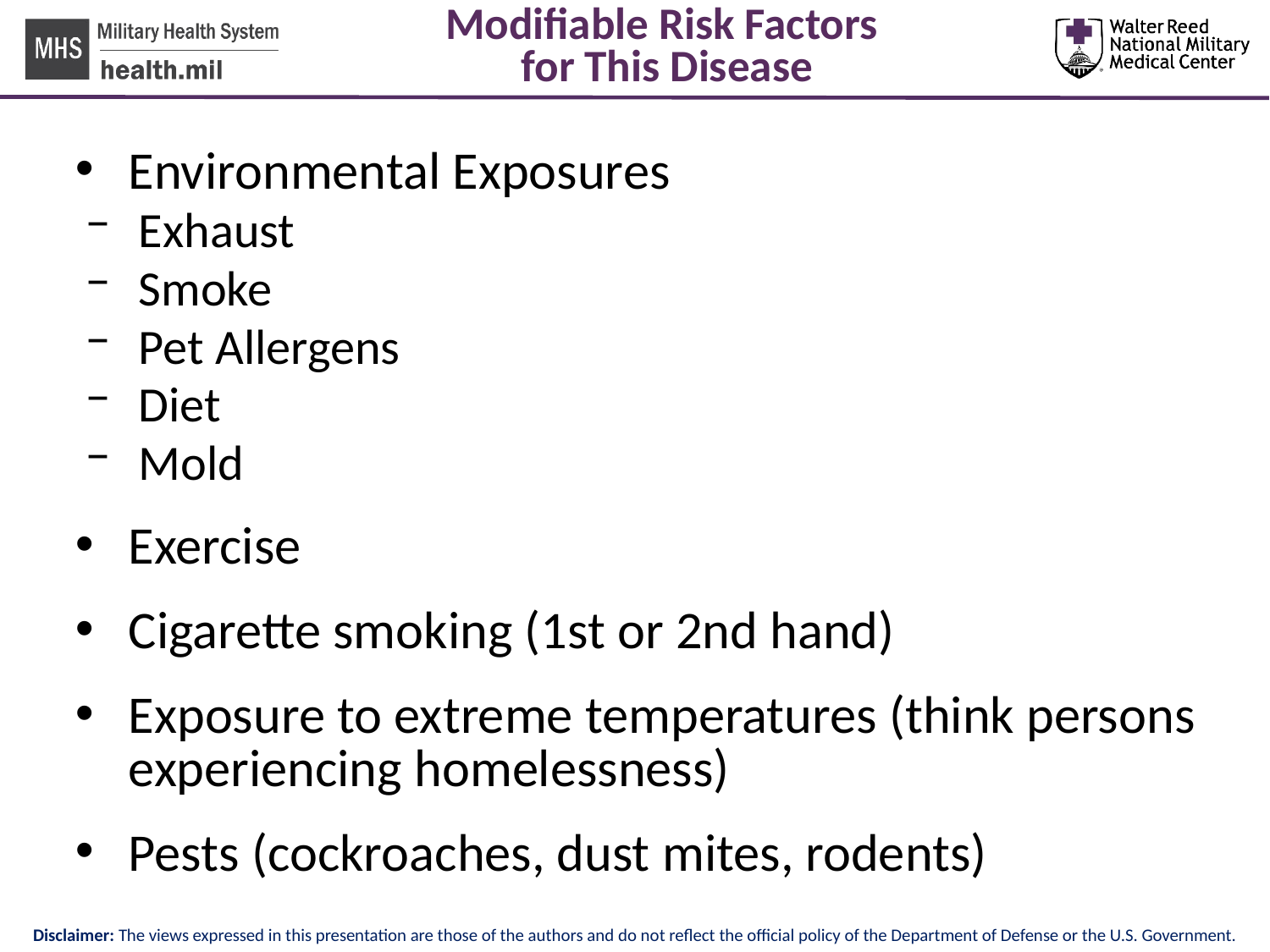

# Modifiable Risk Factors for This Disease
Environmental Exposures
Exhaust
Smoke
Pet Allergens
Diet
Mold
Exercise
Cigarette smoking (1st or 2nd hand)
Exposure to extreme temperatures (think persons experiencing homelessness)
Pests (cockroaches, dust mites, rodents)

## Slide 12
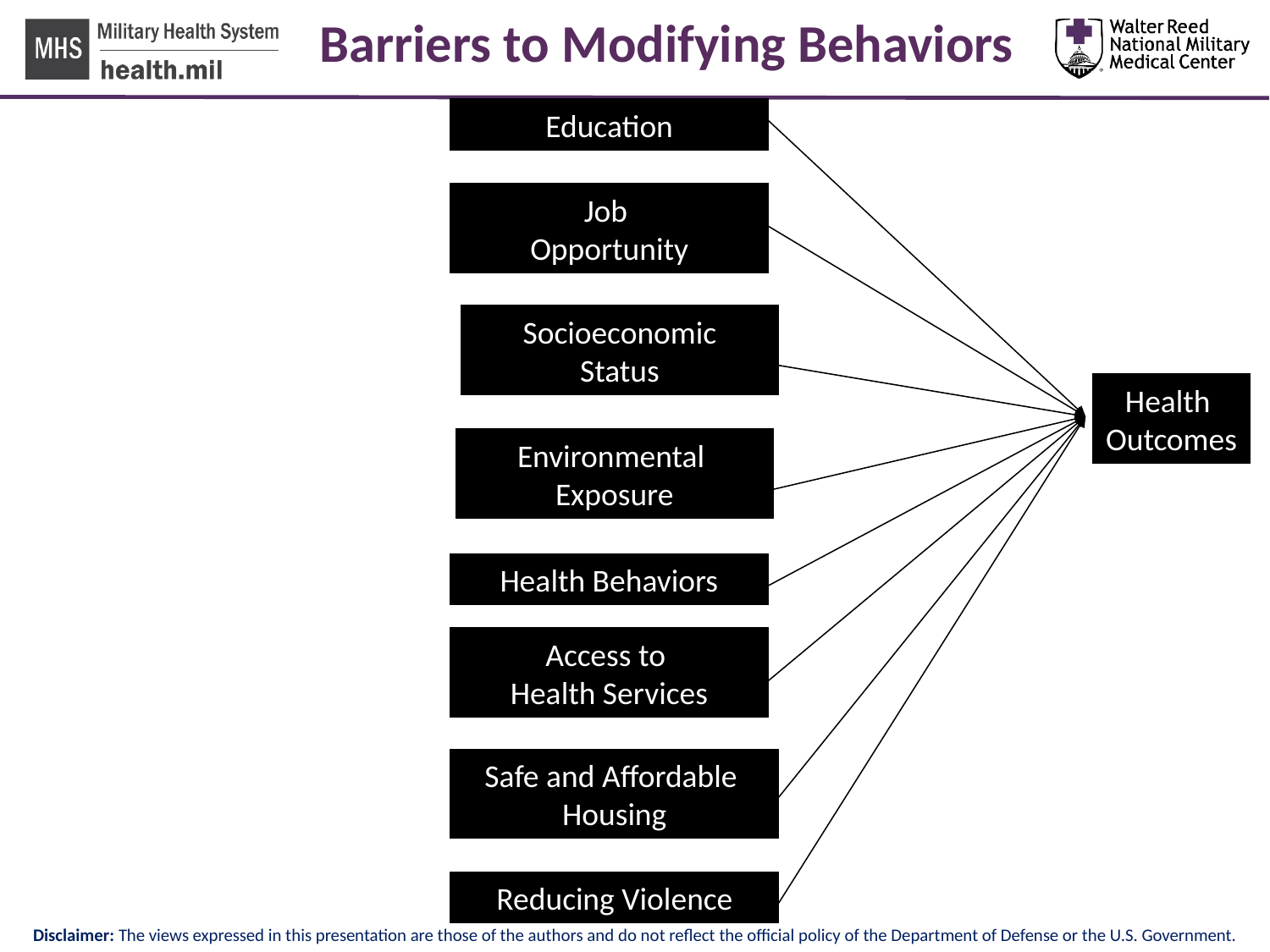

# Barriers to Modifying Behaviors
Education
Job
Opportunity
Socioeconomic
Status
Health
Outcomes
Environmental
Exposure
Health Behaviors
Access to
Health Services
Safe and Affordable
Housing
Reducing Violence

## Slide 13
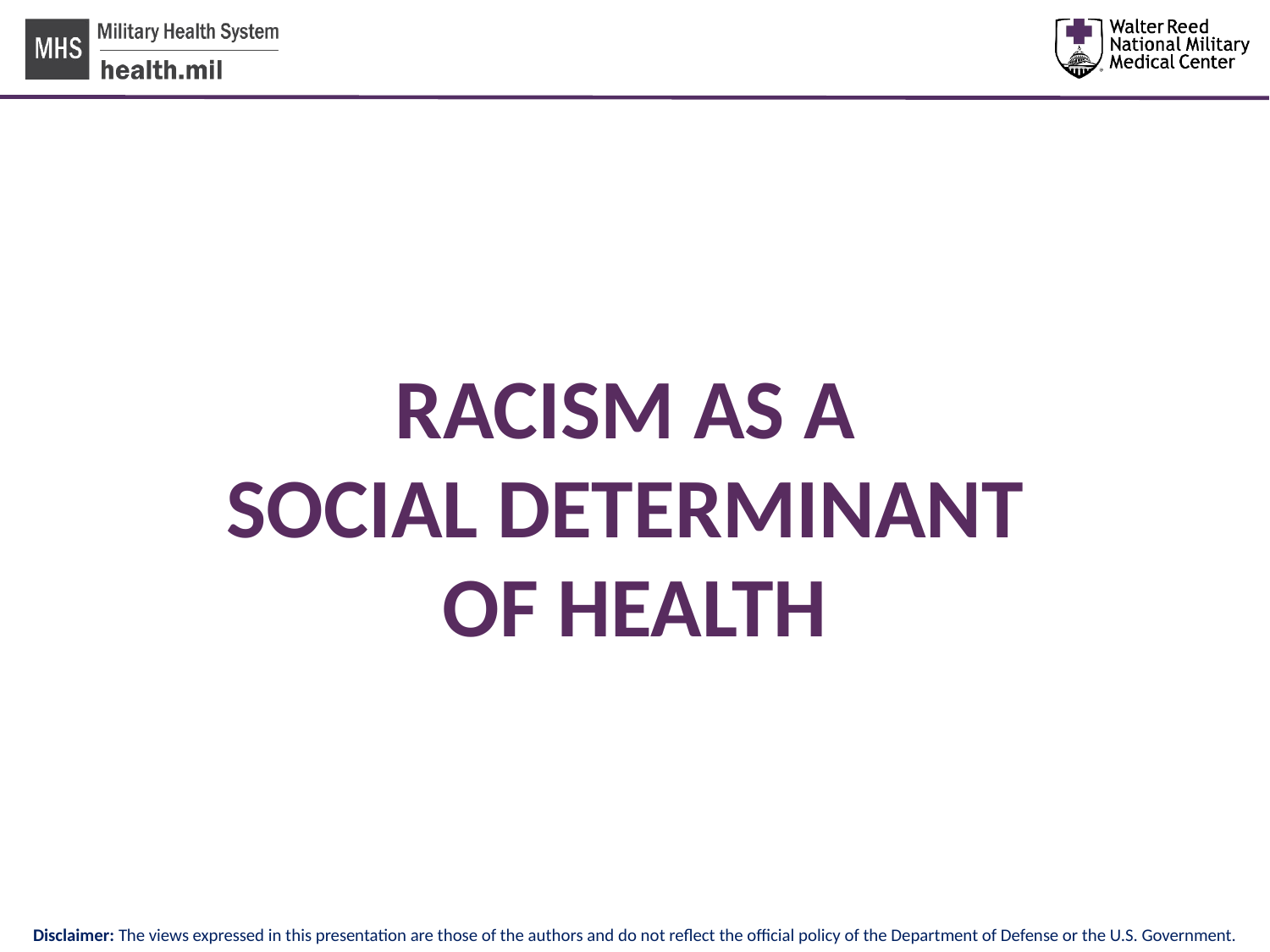

RACISM AS A
SOCIAL DETERMINANT
OF HEALTH

## Slide 14
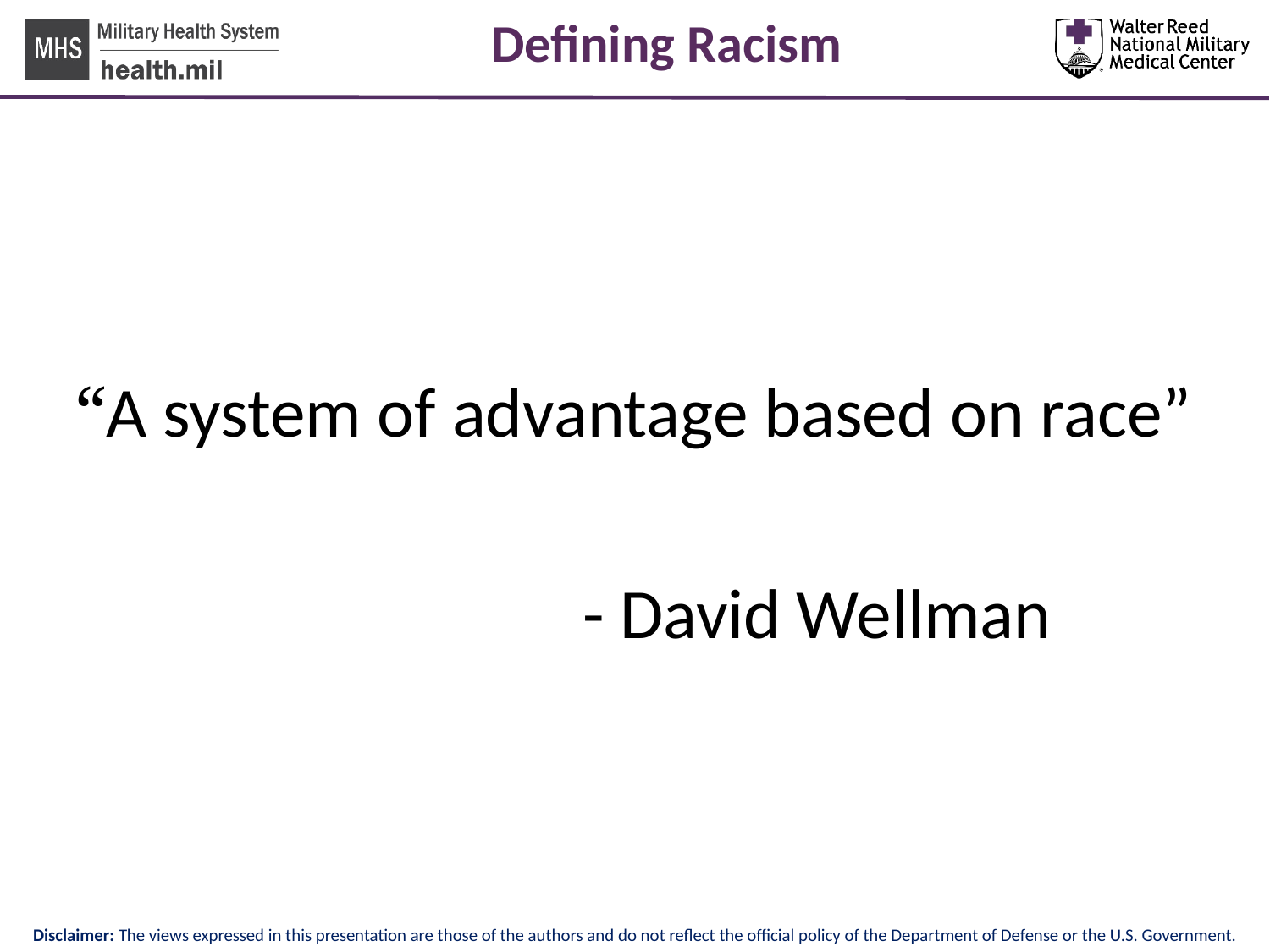

# Defining Racism
	“A system of advantage based on race”
					- David Wellman

## Slide 15
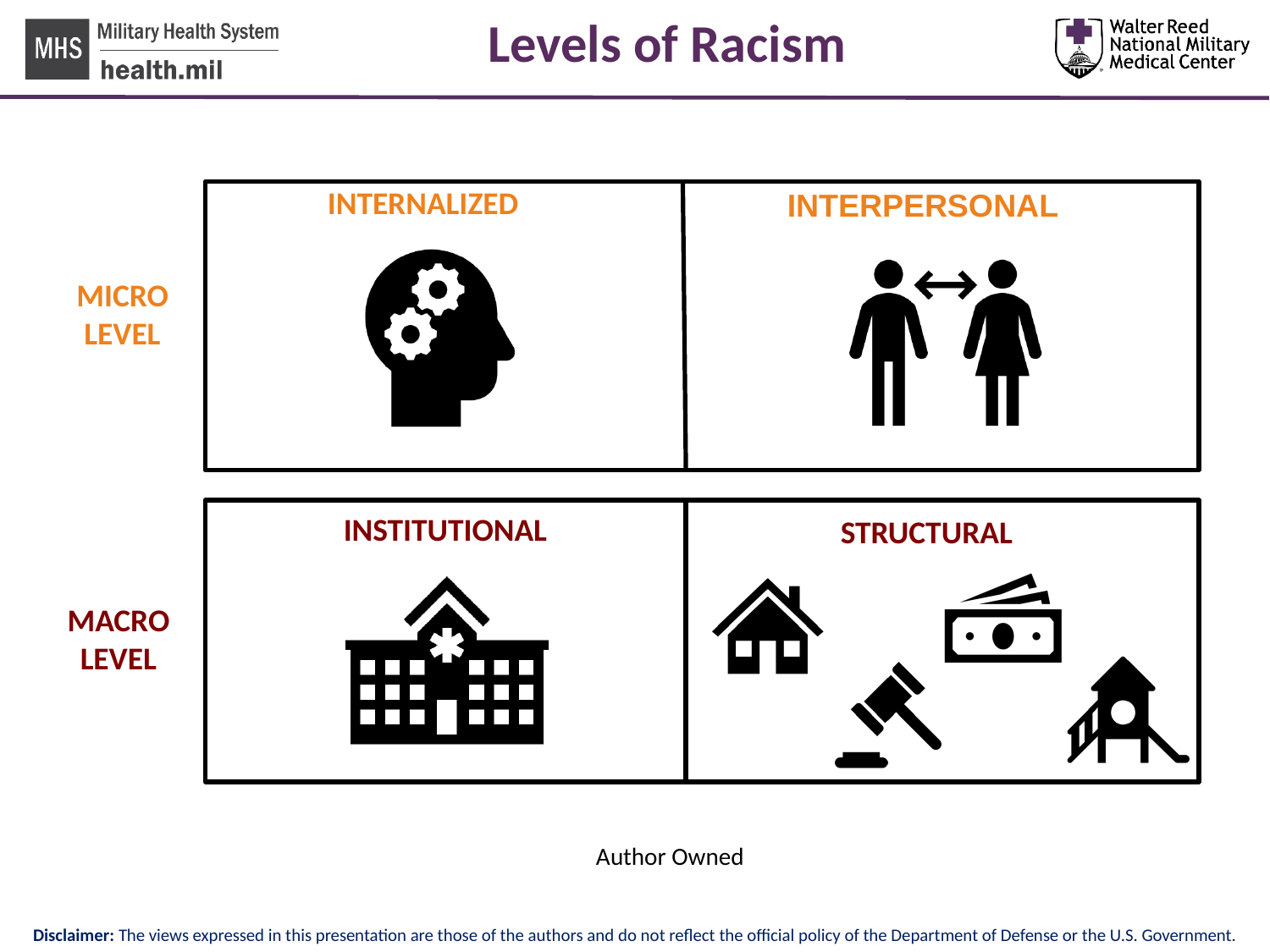

# Levels of Racism
INTERNALIZED
Interpersonal
MICRO LEVEL
INSTITUTIONAL
STRUCTURAL
MACRO LEVEL
Author Owned

## Slide 16
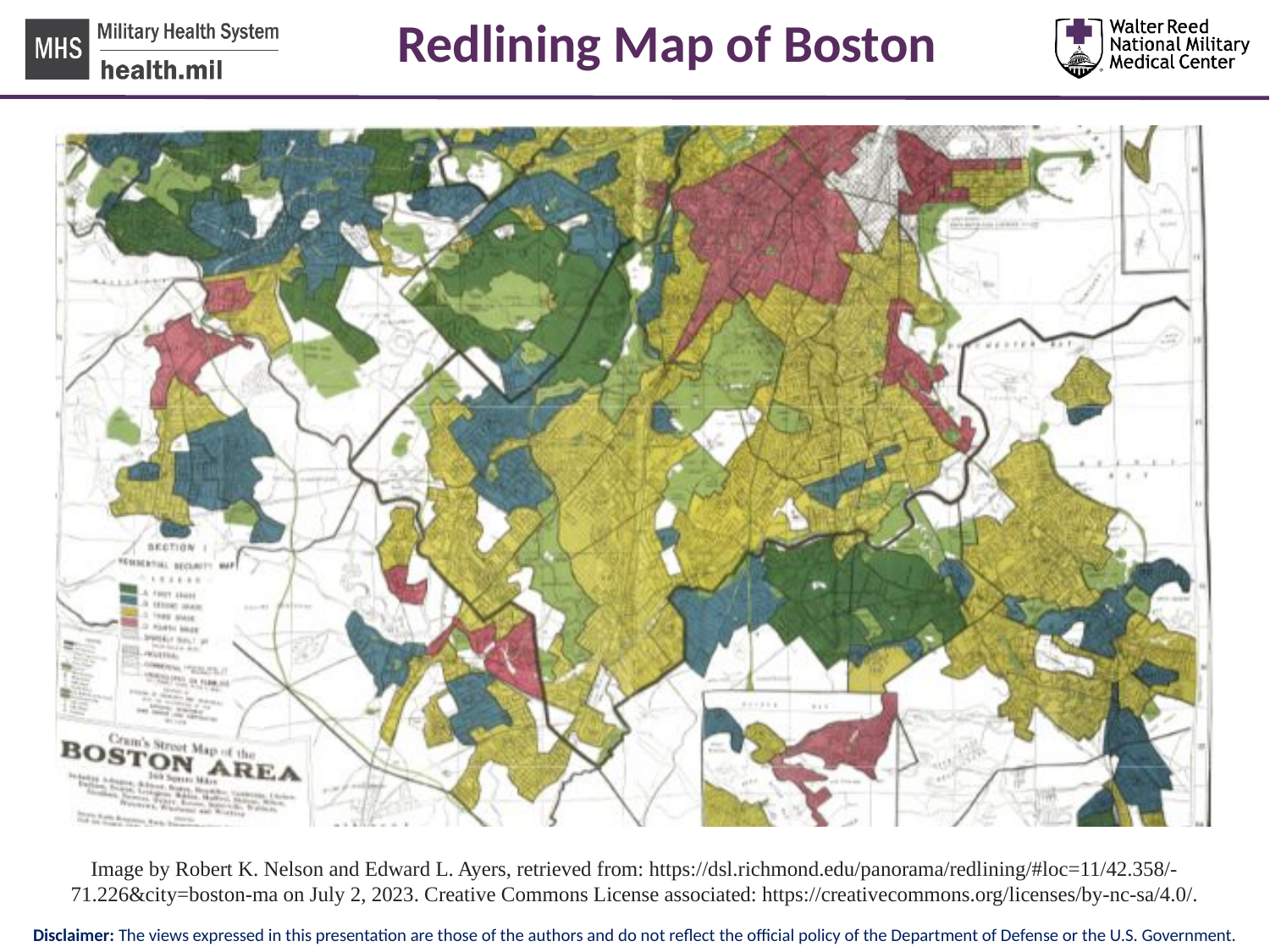

# Redlining Map of Boston
Image by Robert K. Nelson and Edward L. Ayers, retrieved from: https://dsl.richmond.edu/panorama/redlining/#loc=11/42.358/-71.226&city=boston-ma on July 2, 2023. Creative Commons License associated: https://creativecommons.org/licenses/by-nc-sa/4.0/.

## Slide 17
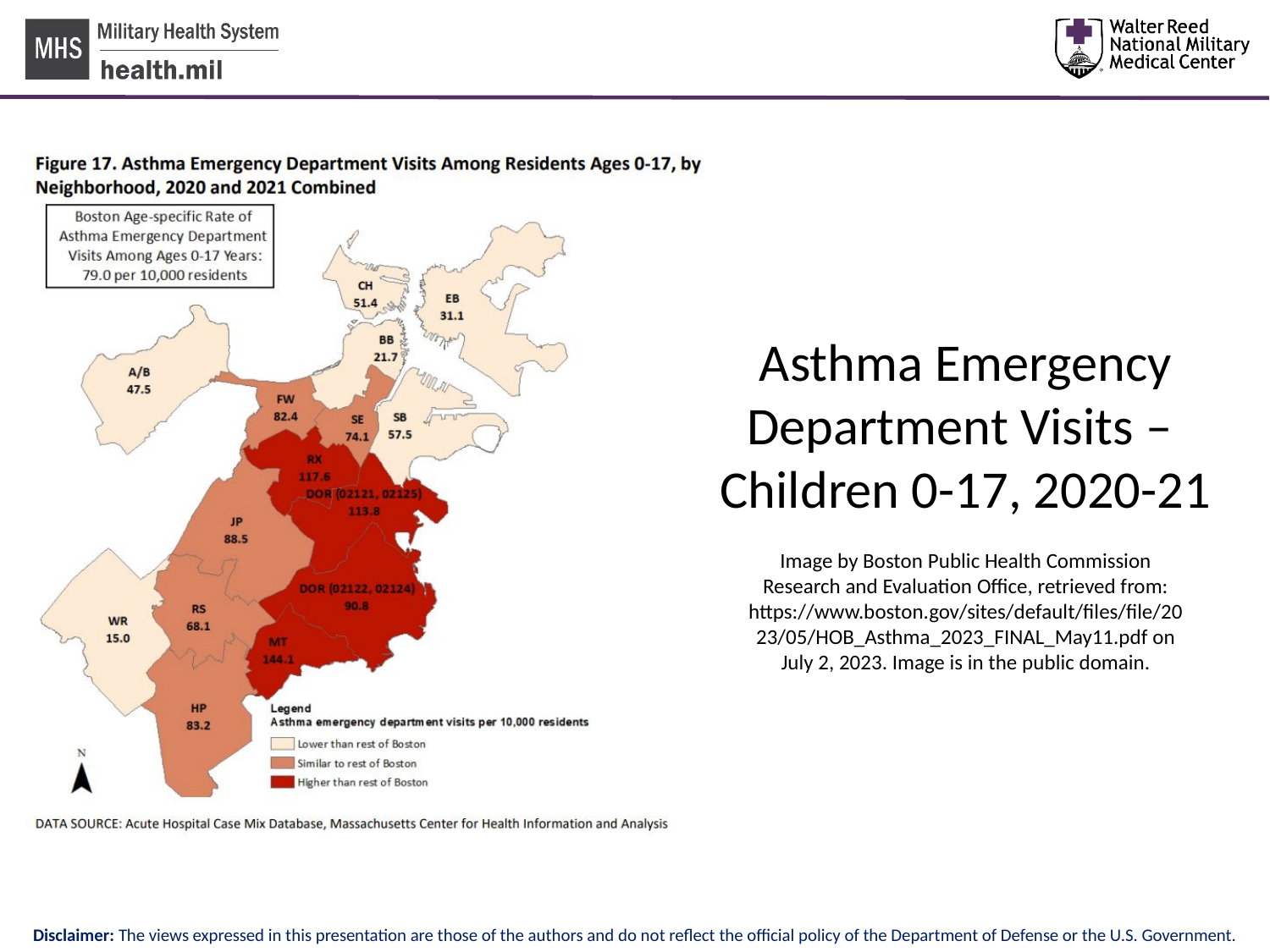

Asthma Emergency Department Visits –
Children 0-17, 2020-21
Image by Boston Public Health Commission Research and Evaluation Office, retrieved from: https://www.boston.gov/sites/default/files/file/2023/05/HOB_Asthma_2023_FINAL_May11.pdf on July 2, 2023. Image is in the public domain.

## Slide 18
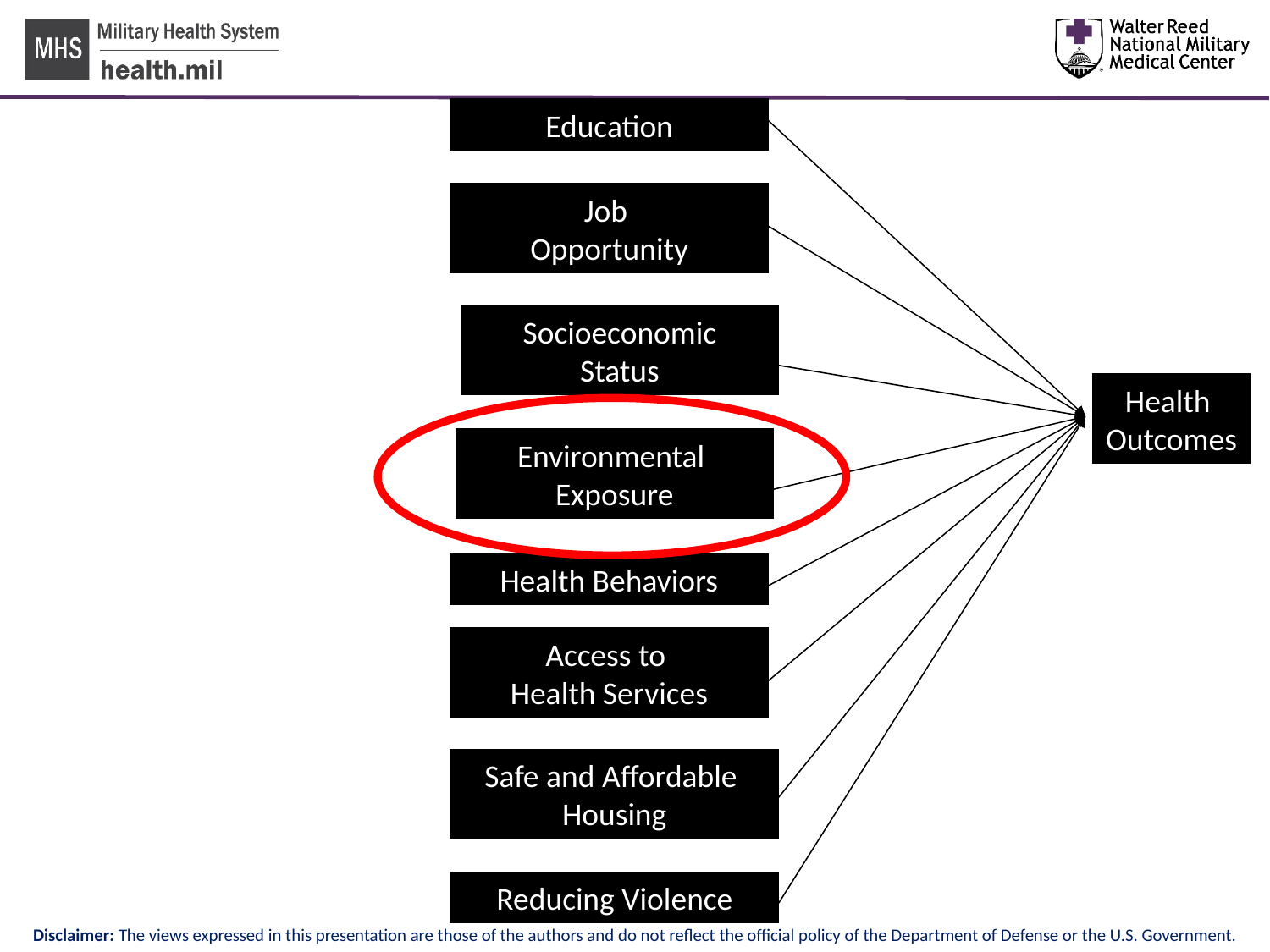

Education
Job
Opportunity
Socioeconomic
Status
Health
Outcomes
Environmental
Exposure
Health Behaviors
Access to
Health Services
Safe and Affordable
Housing
Reducing Violence

## Slide 19
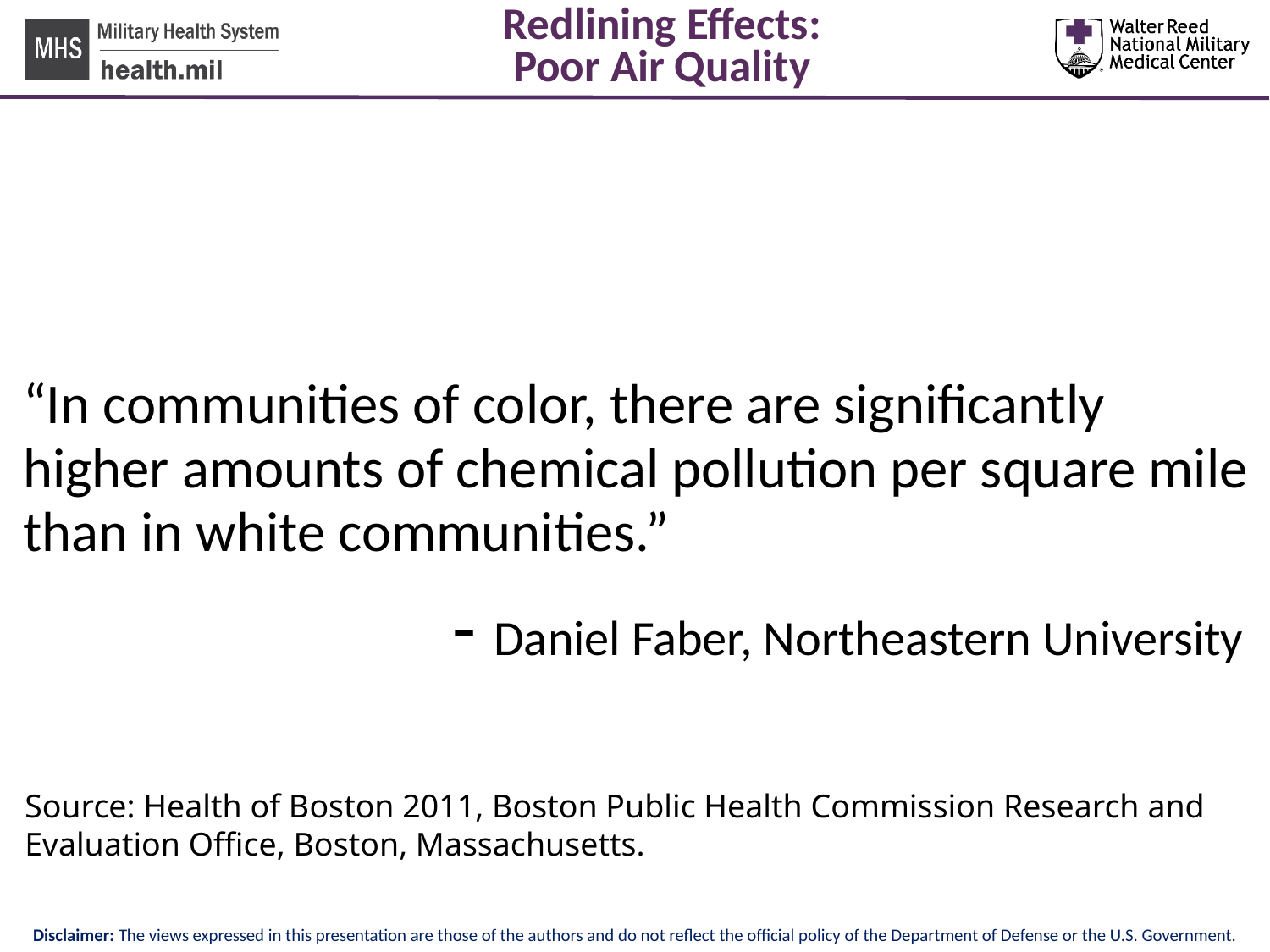

# Redlining Effects: Poor Air Quality
“In communities of color, there are significantly higher amounts of chemical pollution per square mile than in white communities.”
		 - Daniel Faber, Northeastern University
Source: Health of Boston 2011, Boston Public Health Commission Research and Evaluation Office, Boston, Massachusetts.

## Slide 20
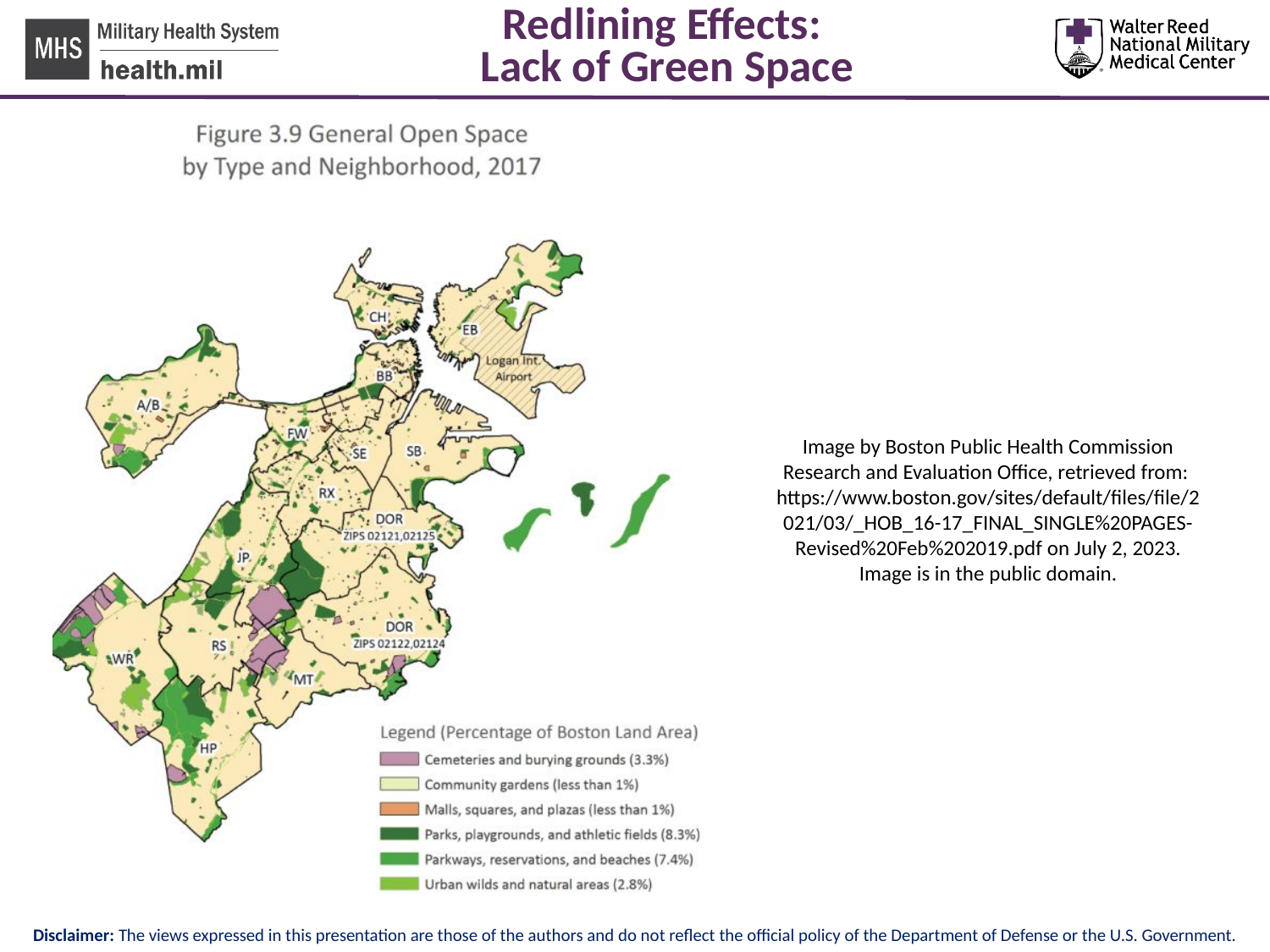

# Redlining Effects: Lack of Green Space
Image by Boston Public Health Commission Research and Evaluation Office, retrieved from: https://www.boston.gov/sites/default/files/file/2021/03/_HOB_16-17_FINAL_SINGLE%20PAGES-Revised%20Feb%202019.pdf on July 2, 2023. Image is in the public domain.

## Slide 21
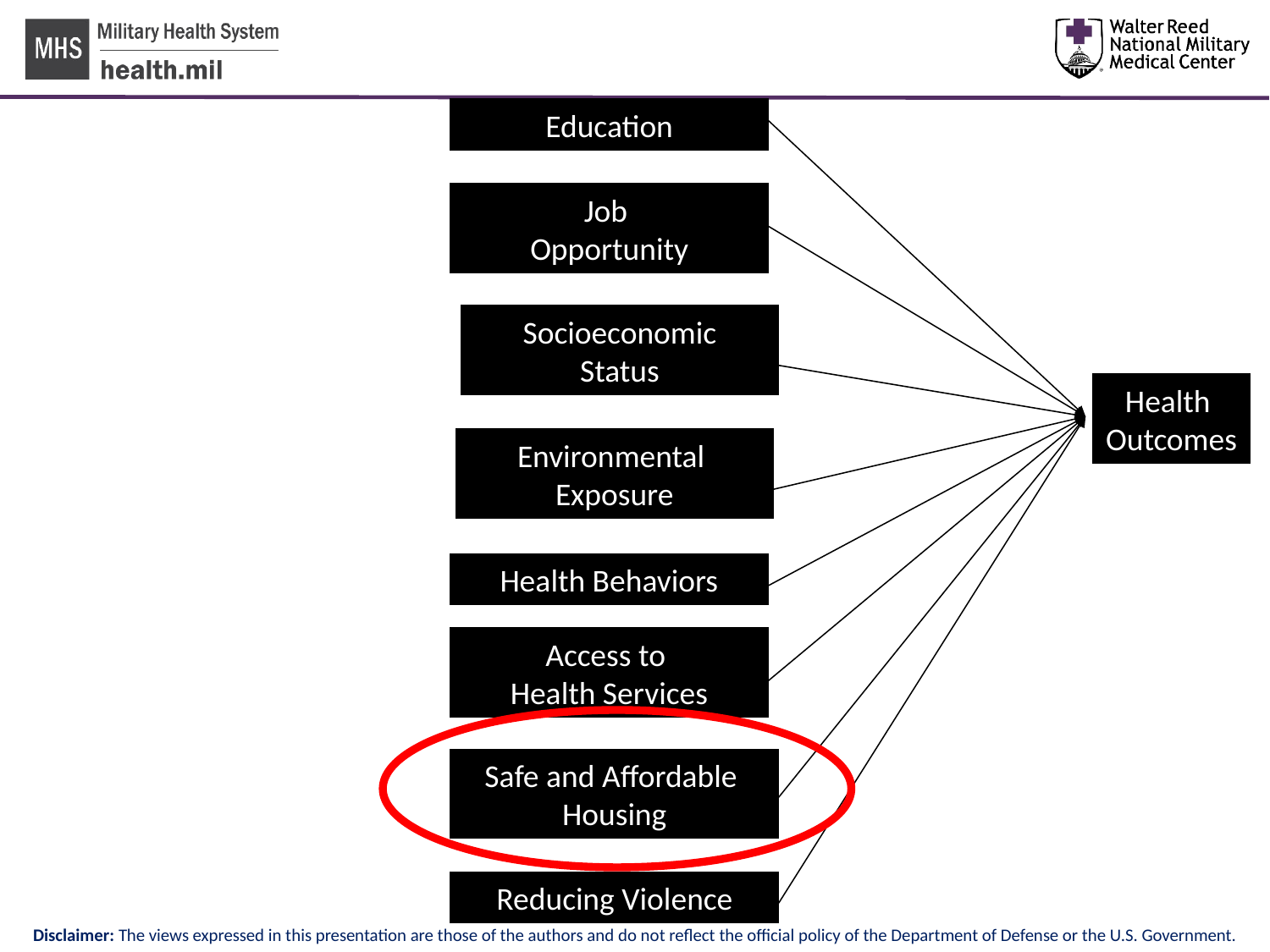

Education
Job
Opportunity
Socioeconomic
Status
Health
Outcomes
Environmental
Exposure
Health Behaviors
Access to
Health Services
Safe and Affordable
Housing
Reducing Violence

## Slide 22
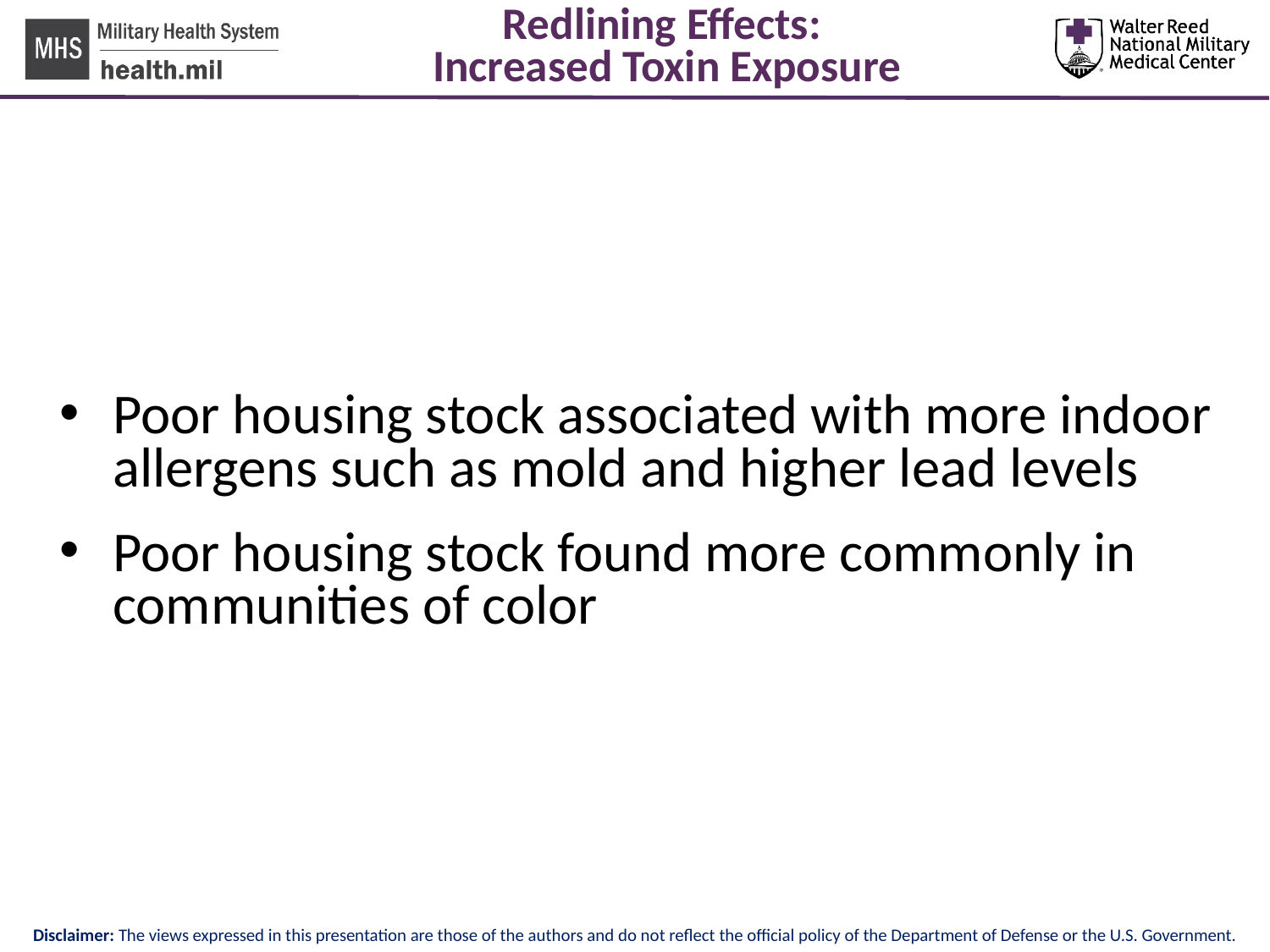

# Redlining Effects: Increased Toxin Exposure
Poor housing stock associated with more indoor allergens such as mold and higher lead levels
Poor housing stock found more commonly in communities of color

## Slide 23
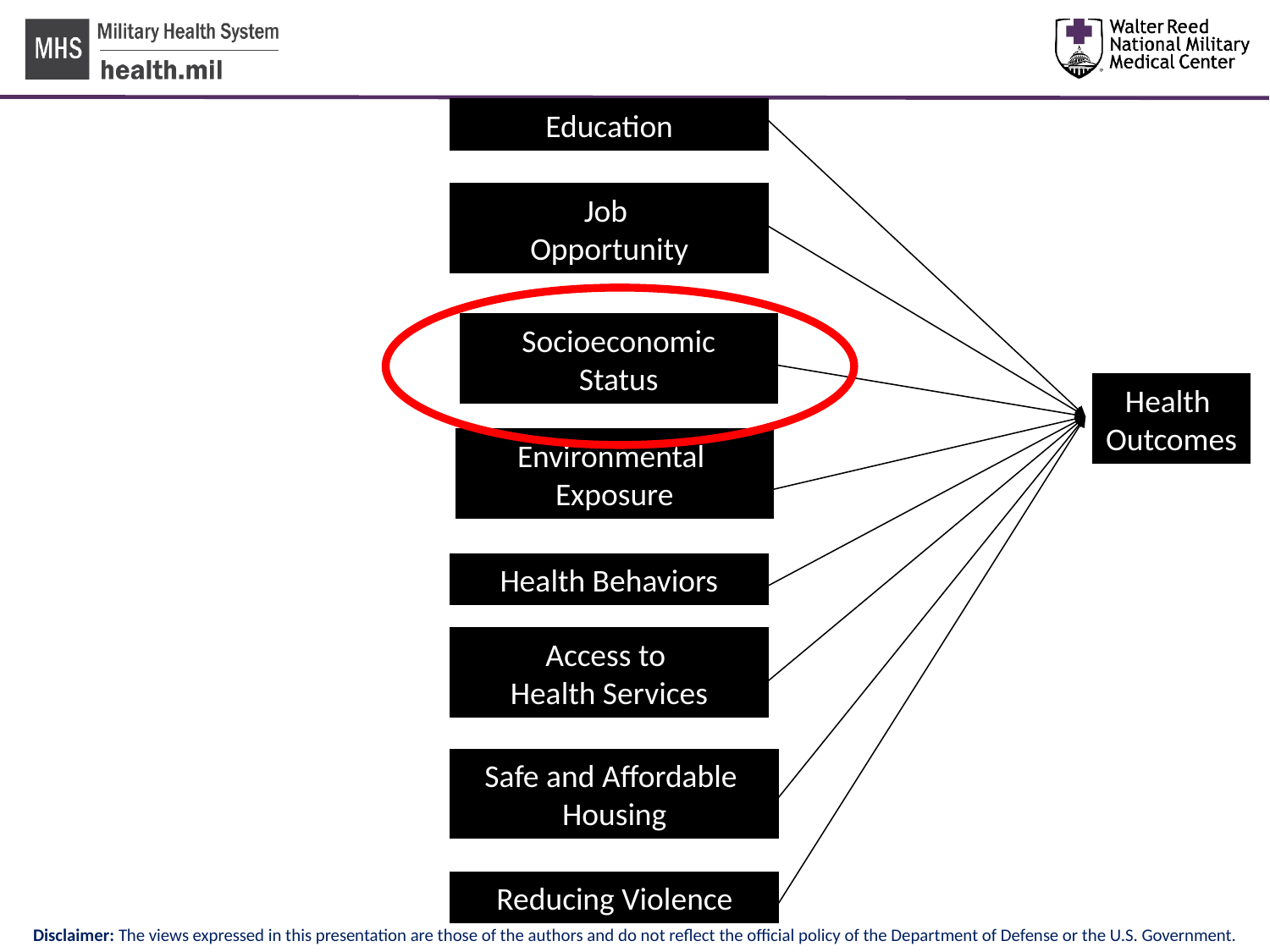

Education
Job
Opportunity
Socioeconomic
Status
Health
Outcomes
Environmental
Exposure
Health Behaviors
Access to
Health Services
Safe and Affordable
Housing
Reducing Violence

## Slide 24
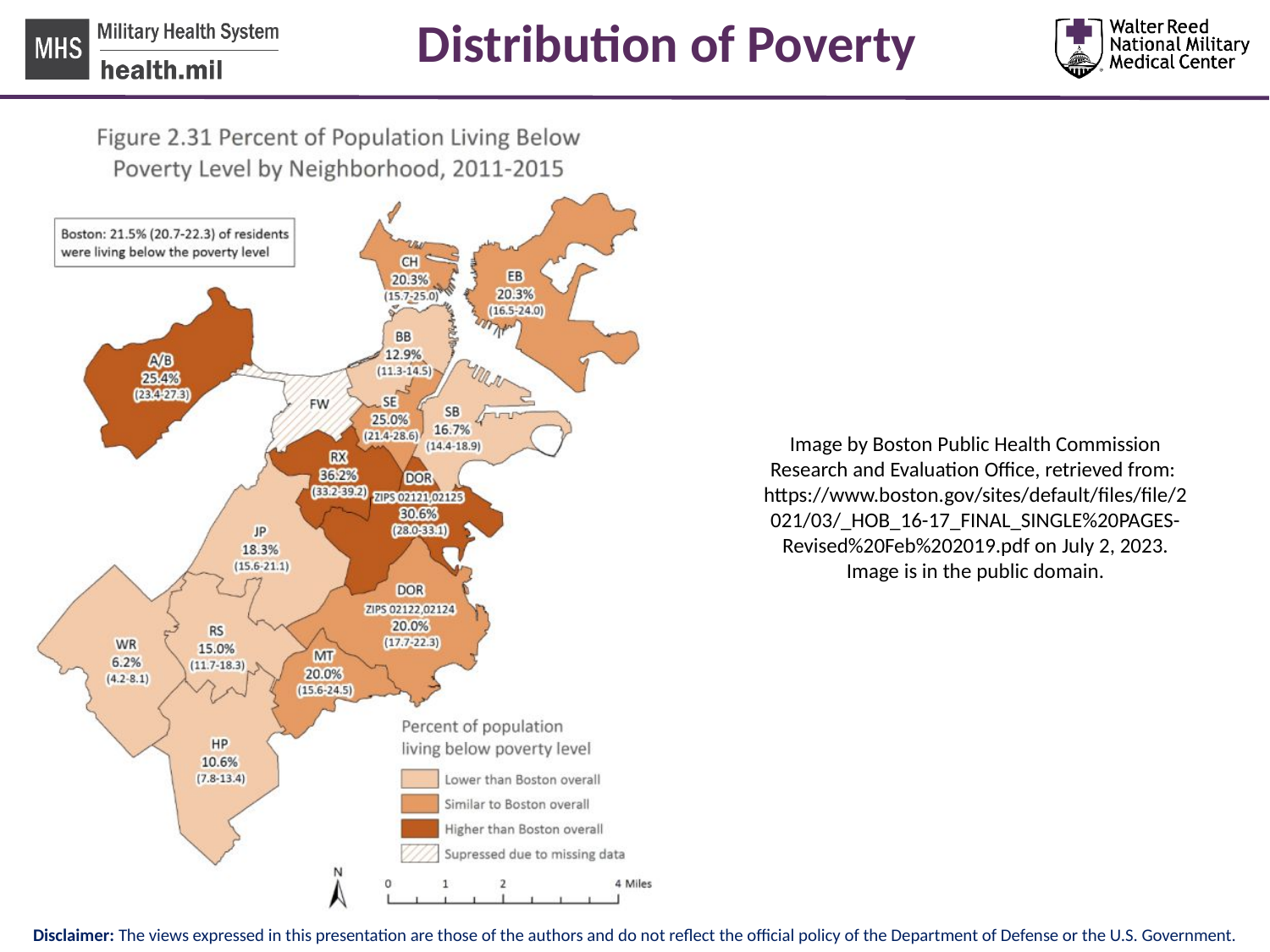

# Distribution of Poverty
Image by Boston Public Health Commission Research and Evaluation Office, retrieved from: https://www.boston.gov/sites/default/files/file/2021/03/_HOB_16-17_FINAL_SINGLE%20PAGES-Revised%20Feb%202019.pdf on July 2, 2023. Image is in the public domain.

## Slide 25
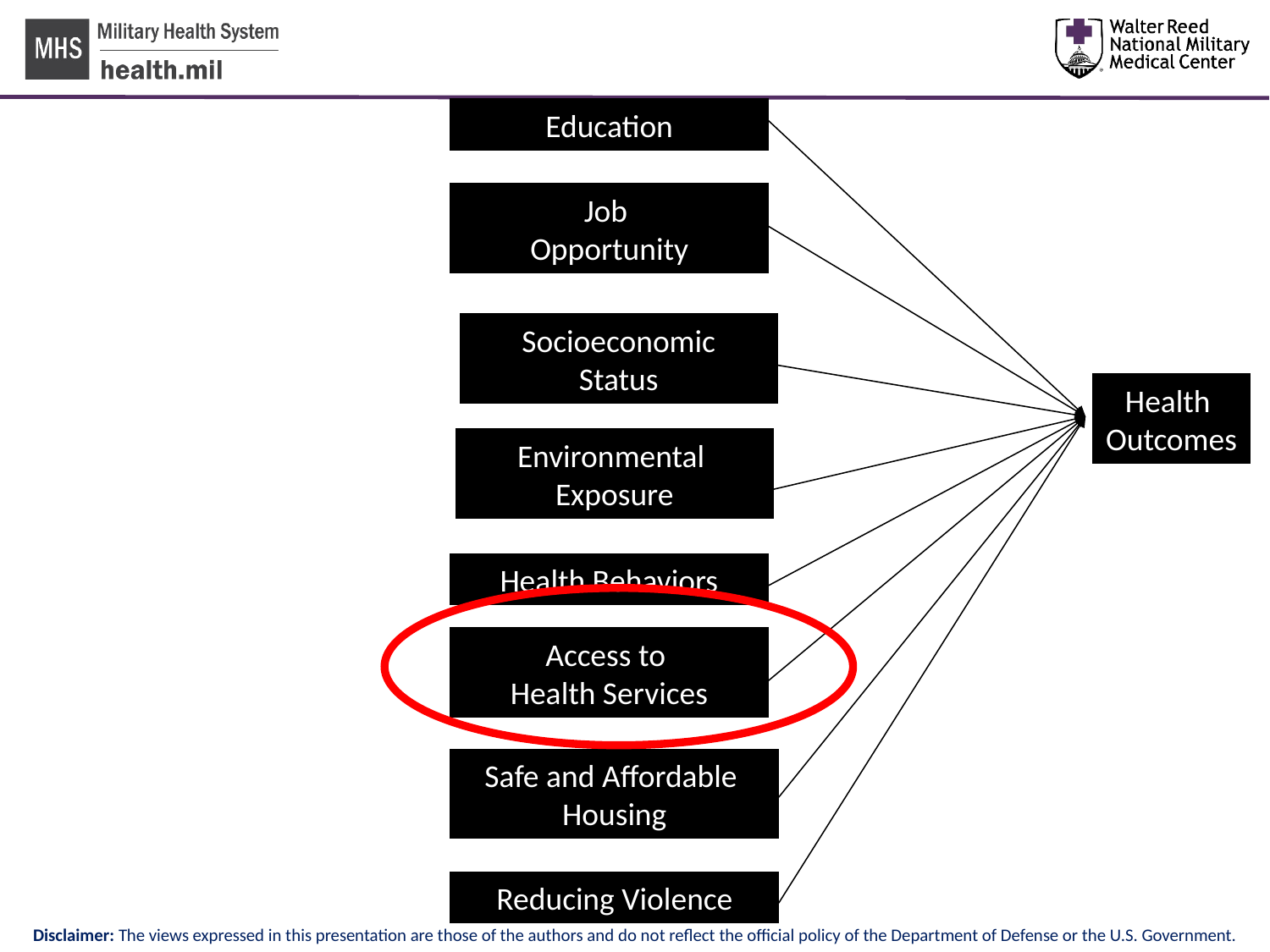

Education
Job
Opportunity
Socioeconomic
Status
Health
Outcomes
Environmental
Exposure
Health Behaviors
Access to
Health Services
Safe and Affordable
Housing
Reducing Violence

## Slide 26
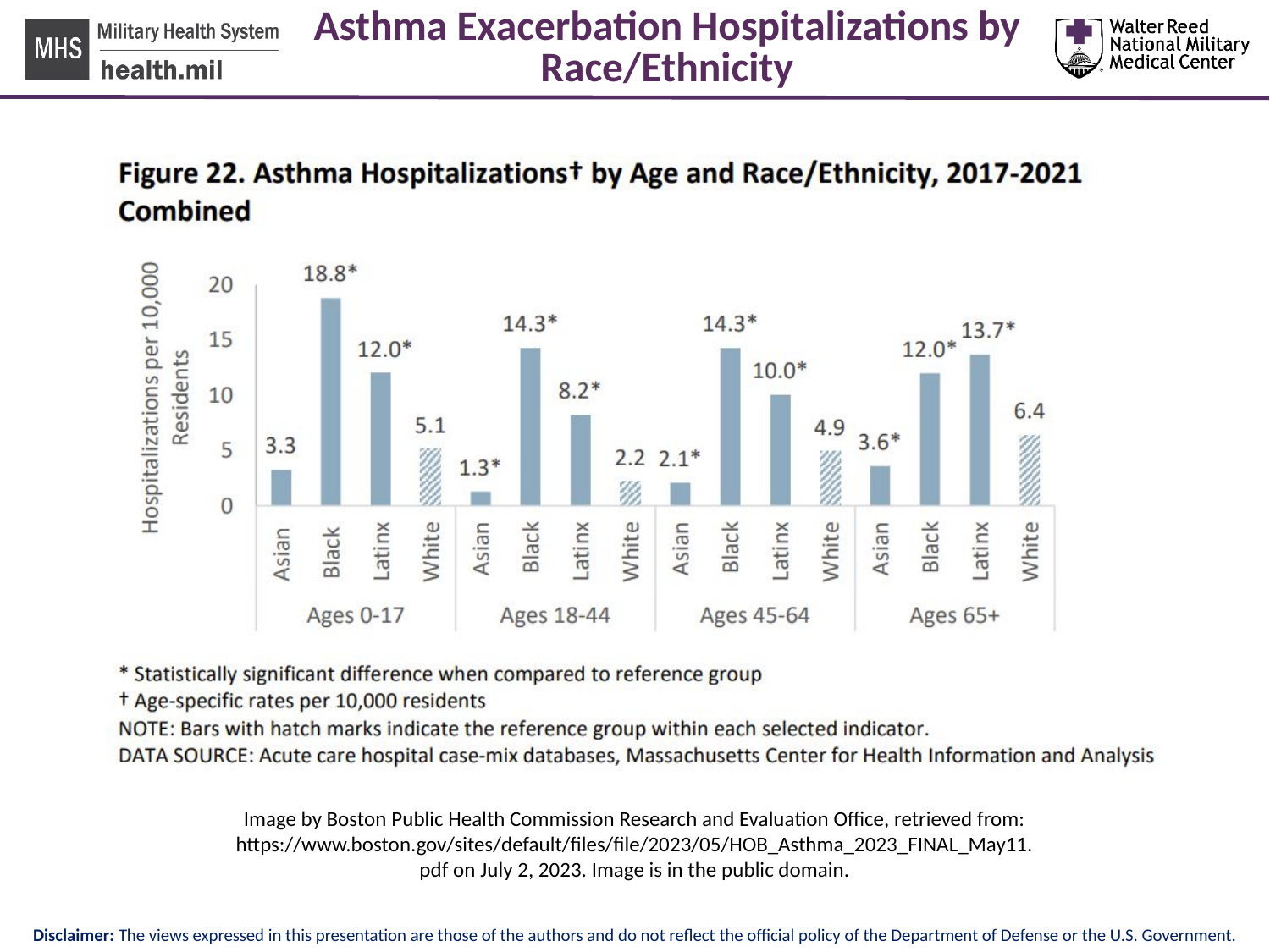

# Asthma Exacerbation Hospitalizations by Race/Ethnicity
Image by Boston Public Health Commission Research and Evaluation Office, retrieved from: https://www.boston.gov/sites/default/files/file/2023/05/HOB_Asthma_2023_FINAL_May11.pdf on July 2, 2023. Image is in the public domain.

## Slide 27
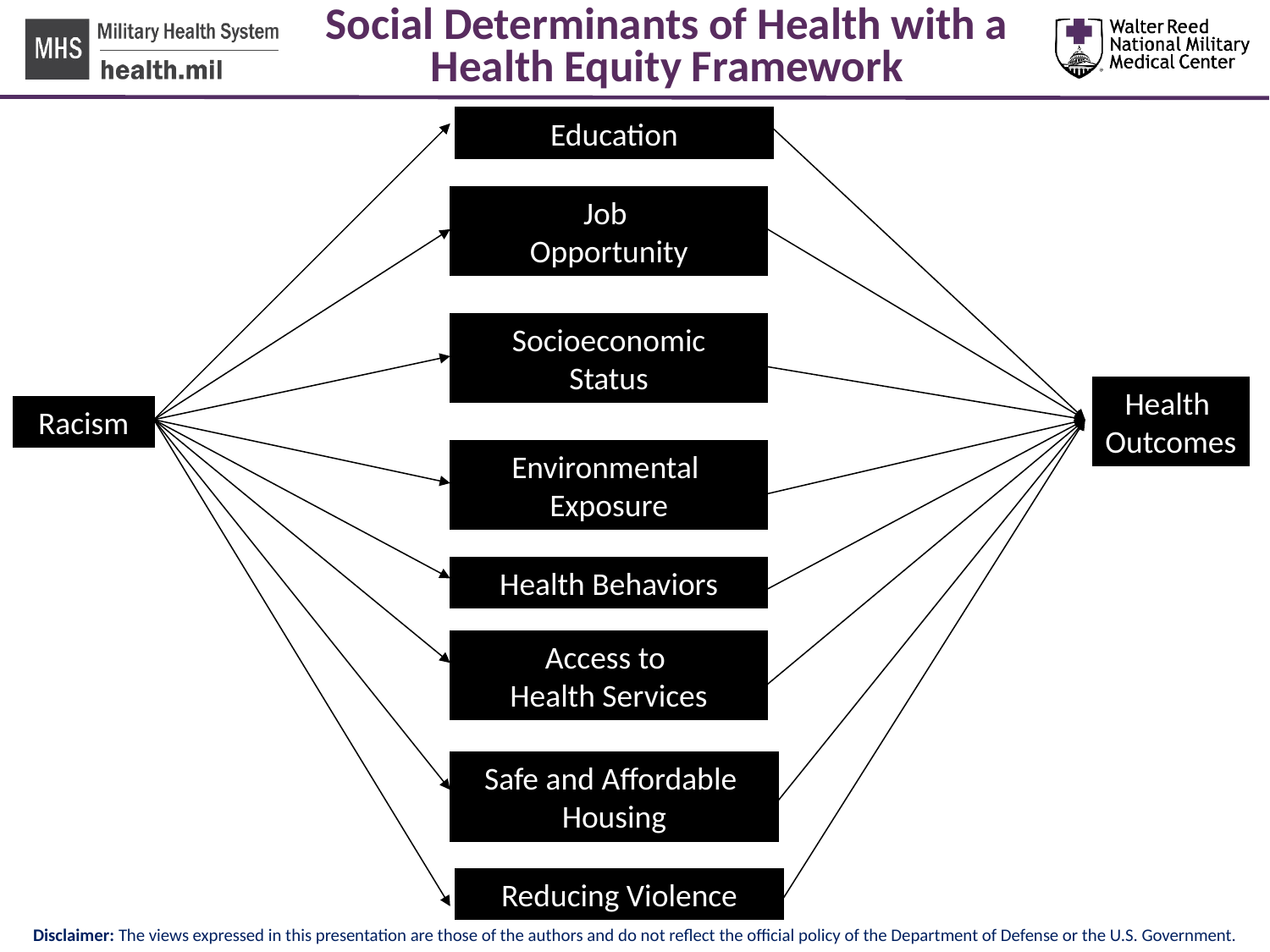

# Social Determinants of Health with a Health Equity Framework
Education
Job
Opportunity
Socioeconomic
Status
Health
Outcomes
Racism
Environmental
Exposure
Health Behaviors
Access to
Health Services
Safe and Affordable
Housing
Reducing Violence

## Slide 28
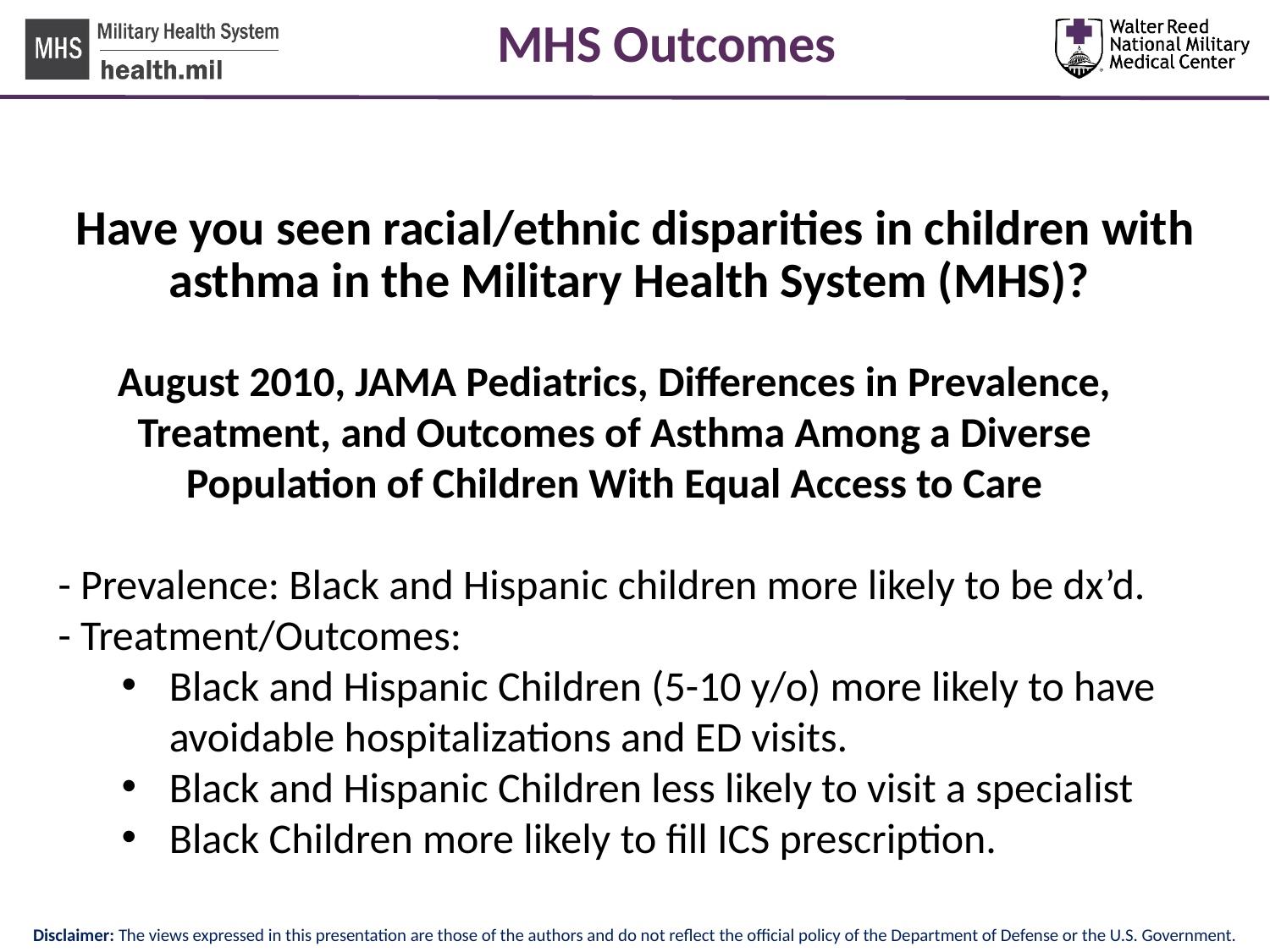

# MHS Outcomes
Have you seen racial/ethnic disparities in children with asthma in the Military Health System (MHS)?
August 2010, JAMA Pediatrics, Differences in Prevalence, Treatment, and Outcomes of Asthma Among a Diverse Population of Children With Equal Access to Care
- Prevalence: Black and Hispanic children more likely to be dx’d.
- Treatment/Outcomes:
Black and Hispanic Children (5-10 y/o) more likely to have avoidable hospitalizations and ED visits.
Black and Hispanic Children less likely to visit a specialist
Black Children more likely to fill ICS prescription.

## Slide 29
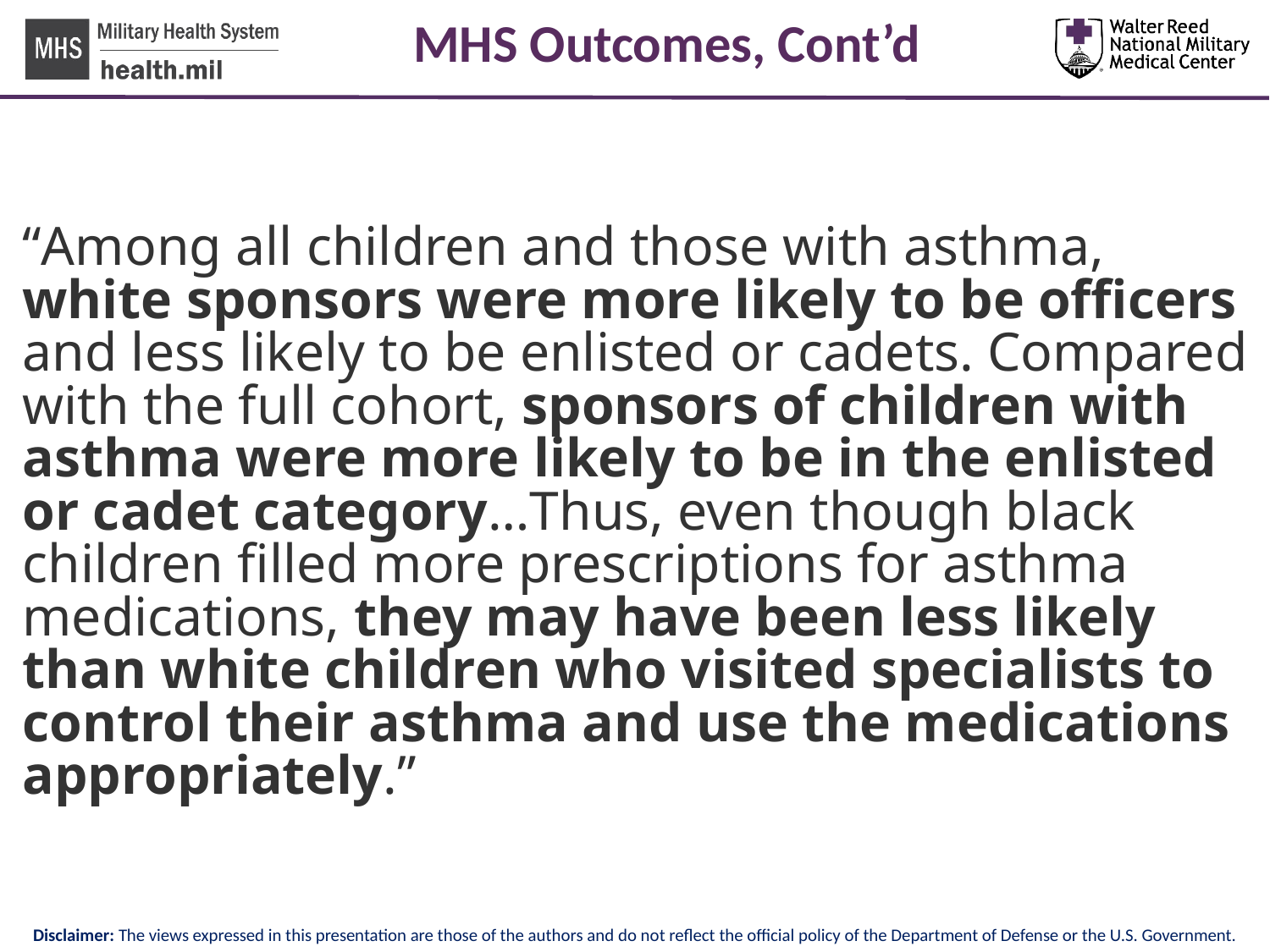

# MHS Outcomes, Cont’d
“Among all children and those with asthma, white sponsors were more likely to be officers and less likely to be enlisted or cadets. Compared with the full cohort, sponsors of children with asthma were more likely to be in the enlisted or cadet category…Thus, even though black children filled more prescriptions for asthma medications, they may have been less likely than white children who visited specialists to control their asthma and use the medications appropriately.”

## Slide 30
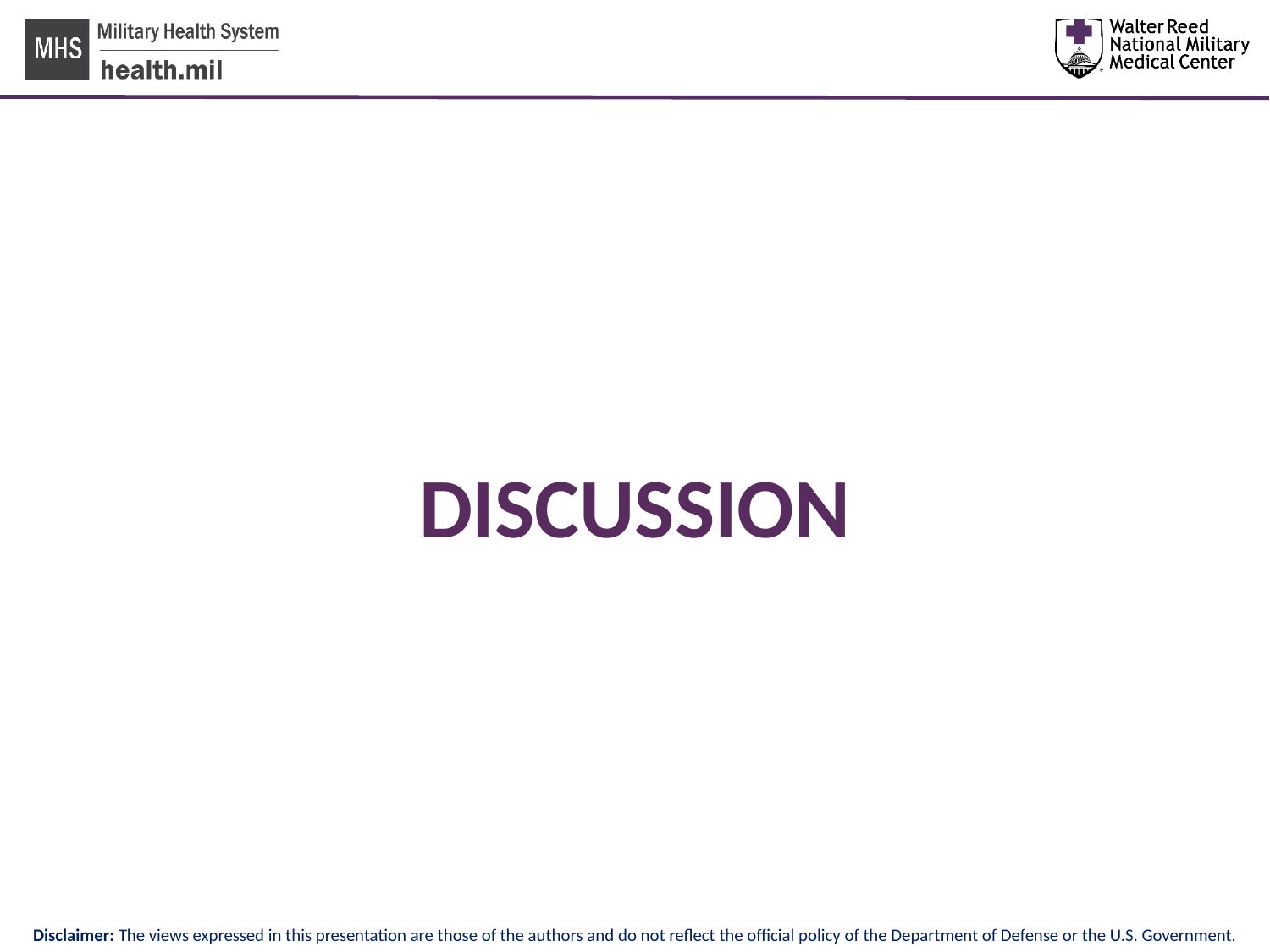

DISCUSSION

## Slide 31
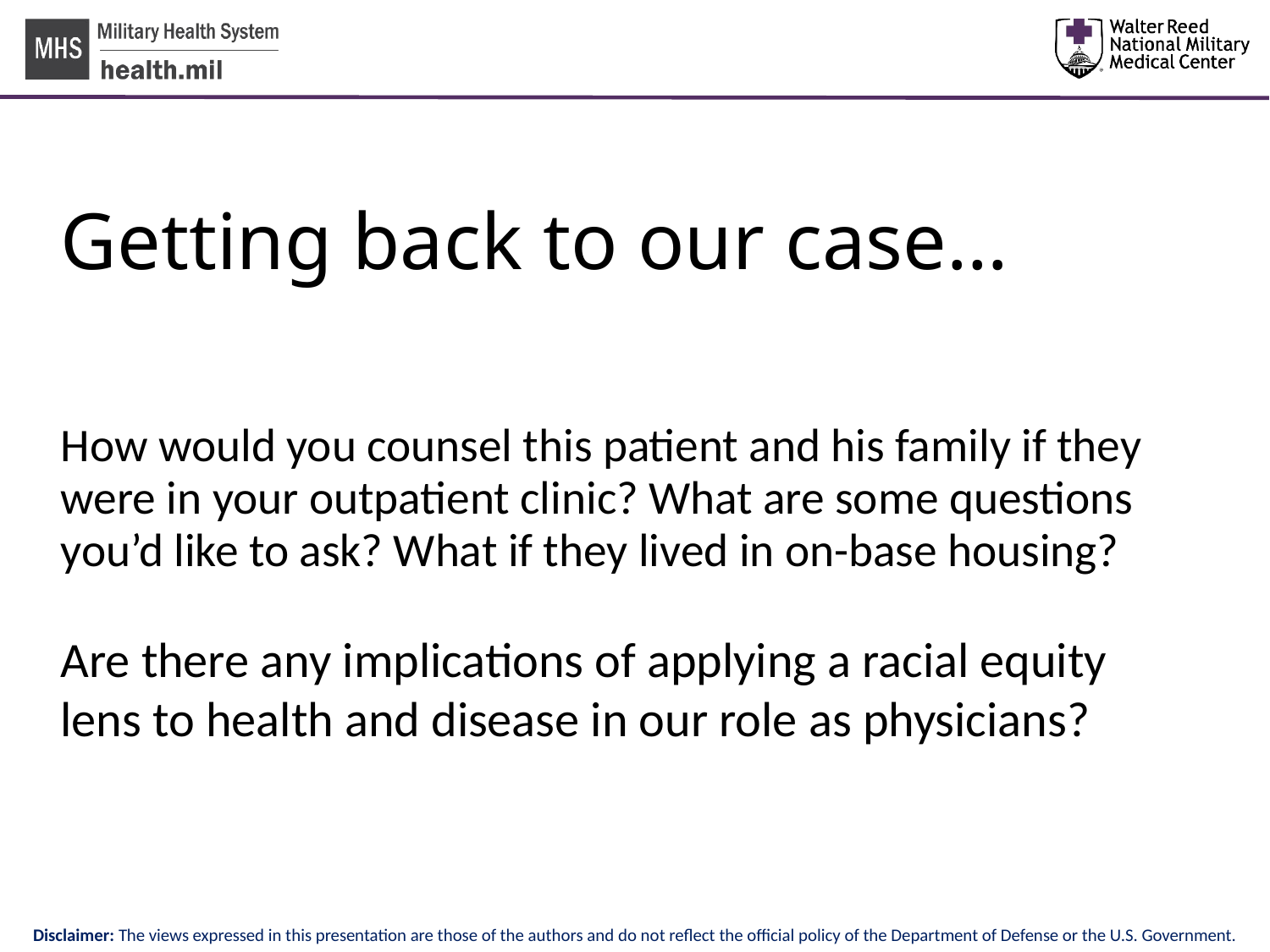

Getting back to our case…
How would you counsel this patient and his family if they were in your outpatient clinic? What are some questions you’d like to ask? What if they lived in on-base housing?
Are there any implications of applying a racial equity lens to health and disease in our role as physicians?

## Slide 32
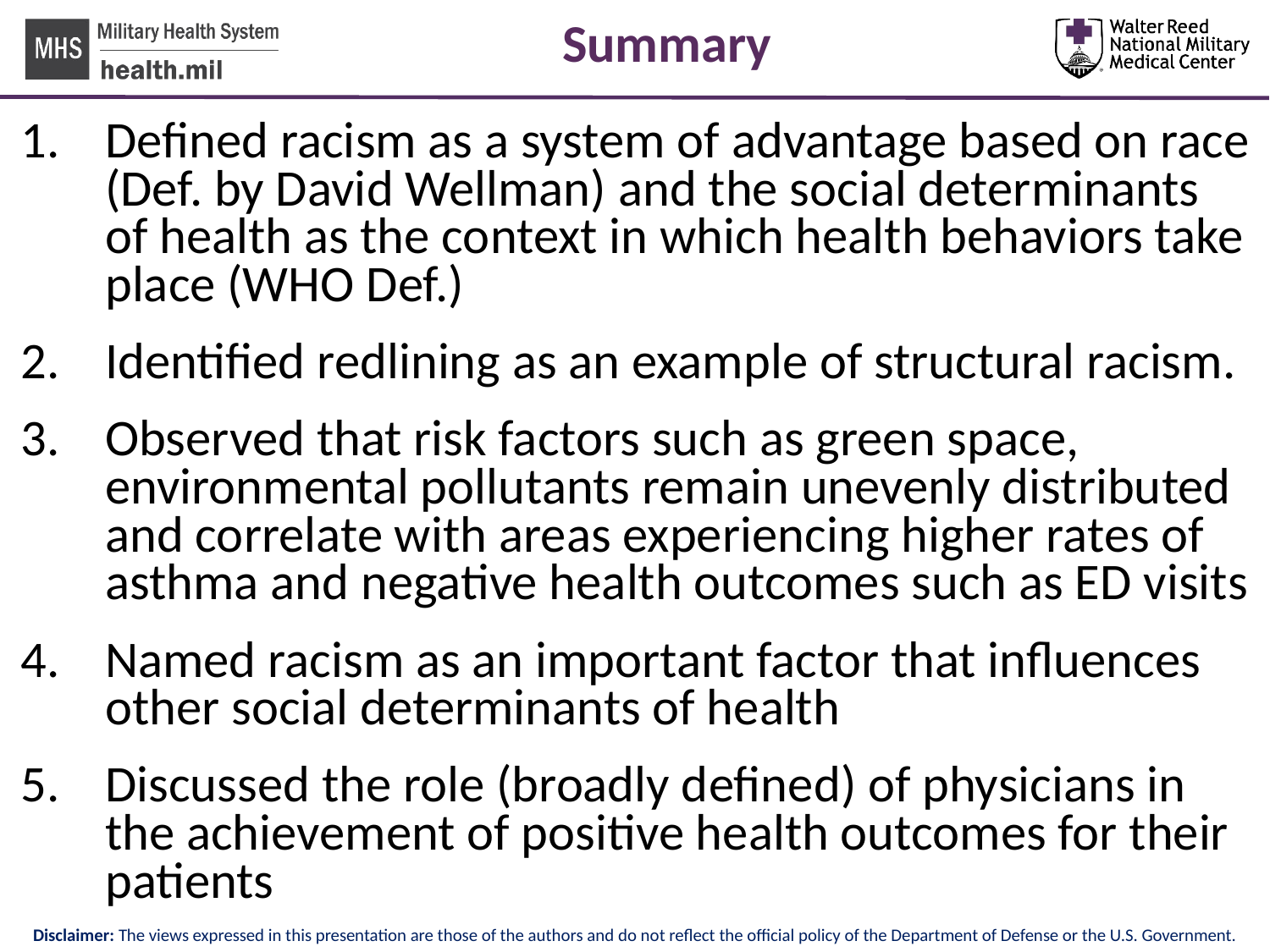

# Summary
Defined racism as a system of advantage based on race (Def. by David Wellman) and the social determinants of health as the context in which health behaviors take place (WHO Def.)
Identified redlining as an example of structural racism.
Observed that risk factors such as green space, environmental pollutants remain unevenly distributed and correlate with areas experiencing higher rates of asthma and negative health outcomes such as ED visits
Named racism as an important factor that influences other social determinants of health
Discussed the role (broadly defined) of physicians in the achievement of positive health outcomes for their patients

## Slide 33
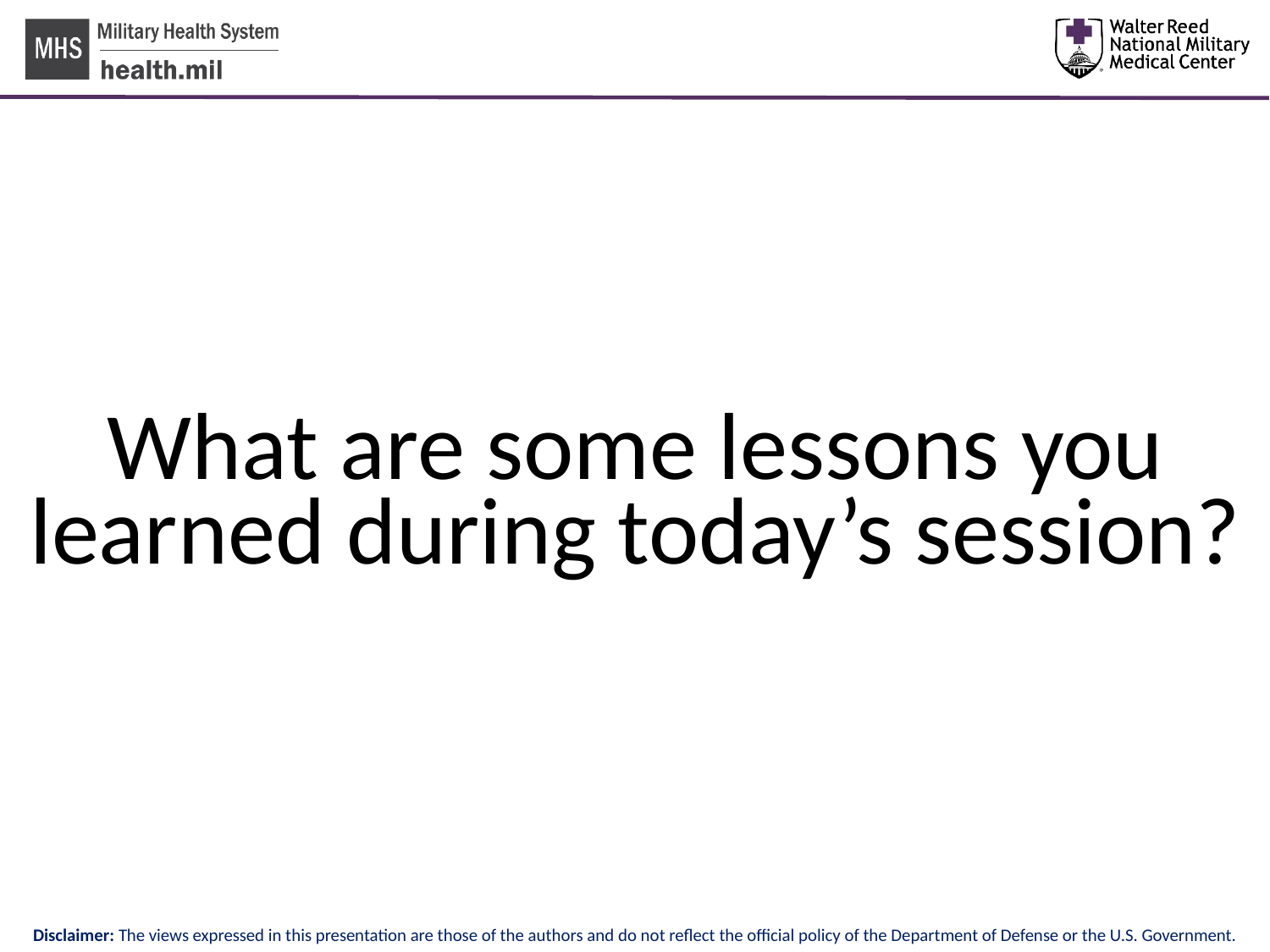

What are some lessons you
learned during today’s session?

## Slide 34
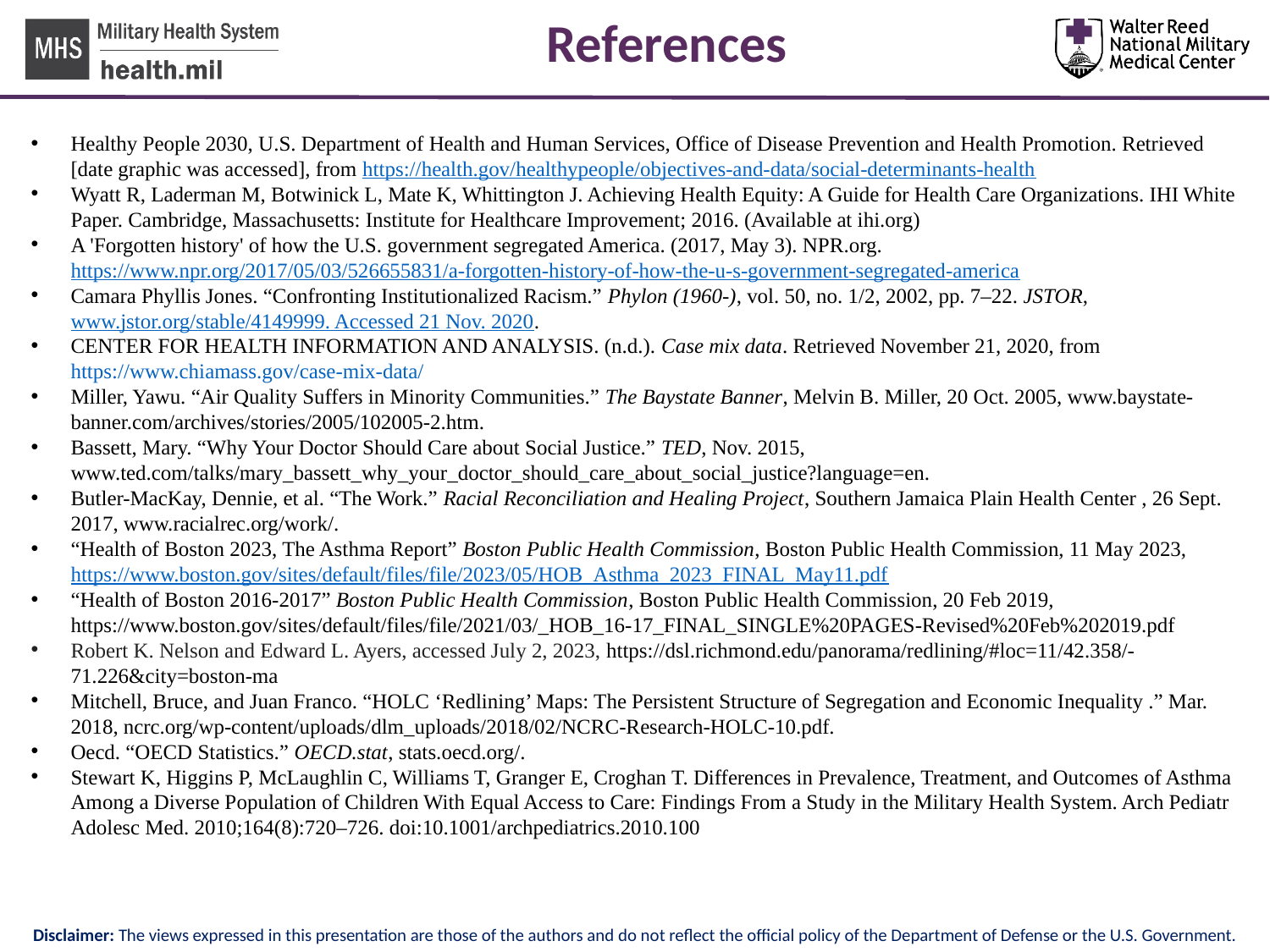

# References
Healthy People 2030, U.S. Department of Health and Human Services, Office of Disease Prevention and Health Promotion. Retrieved [date graphic was accessed], from https://health.gov/healthypeople/objectives-and-data/social-determinants-health
Wyatt R, Laderman M, Botwinick L, Mate K, Whittington J. Achieving Health Equity: A Guide for Health Care Organizations. IHI White Paper. Cambridge, Massachusetts: Institute for Healthcare Improvement; 2016. (Available at ihi.org)
A 'Forgotten history' of how the U.S. government segregated America. (2017, May 3). NPR.org. https://www.npr.org/2017/05/03/526655831/a-forgotten-history-of-how-the-u-s-government-segregated-america
Camara Phyllis Jones. “Confronting Institutionalized Racism.” Phylon (1960-), vol. 50, no. 1/2, 2002, pp. 7–22. JSTOR, www.jstor.org/stable/4149999. Accessed 21 Nov. 2020.
CENTER FOR HEALTH INFORMATION AND ANALYSIS. (n.d.). Case mix data. Retrieved November 21, 2020, from https://www.chiamass.gov/case-mix-data/
Miller, Yawu. “Air Quality Suffers in Minority Communities.” The Baystate Banner, Melvin B. Miller, 20 Oct. 2005, www.baystate-banner.com/archives/stories/2005/102005-2.htm.
Bassett, Mary. “Why Your Doctor Should Care about Social Justice.” TED, Nov. 2015, www.ted.com/talks/mary_bassett_why_your_doctor_should_care_about_social_justice?language=en.
Butler-MacKay, Dennie, et al. “The Work.” Racial Reconciliation and Healing Project, Southern Jamaica Plain Health Center , 26 Sept. 2017, www.racialrec.org/work/.
“Health of Boston 2023, The Asthma Report” Boston Public Health Commission, Boston Public Health Commission, 11 May 2023, https://www.boston.gov/sites/default/files/file/2023/05/HOB_Asthma_2023_FINAL_May11.pdf
“Health of Boston 2016-2017” Boston Public Health Commission, Boston Public Health Commission, 20 Feb 2019, https://www.boston.gov/sites/default/files/file/2021/03/_HOB_16-17_FINAL_SINGLE%20PAGES-Revised%20Feb%202019.pdf
Robert K. Nelson and Edward L. Ayers, accessed July 2, 2023, https://dsl.richmond.edu/panorama/redlining/#loc=11/42.358/-71.226&city=boston-ma
Mitchell, Bruce, and Juan Franco. “HOLC ‘Redlining’ Maps: The Persistent Structure of Segregation and Economic Inequality .” Mar. 2018, ncrc.org/wp-content/uploads/dlm_uploads/2018/02/NCRC-Research-HOLC-10.pdf.
Oecd. “OECD Statistics.” OECD.stat, stats.oecd.org/.
Stewart K, Higgins P, McLaughlin C, Williams T, Granger E, Croghan T. Differences in Prevalence, Treatment, and Outcomes of Asthma Among a Diverse Population of Children With Equal Access to Care: Findings From a Study in the Military Health System. Arch Pediatr Adolesc Med. 2010;164(8):720–726. doi:10.1001/archpediatrics.2010.100
