# Supplementary material for: The Impact of Racism on Health: A Health Equity Training on Structural Racism for Military Residents and Fellows
Source: MedEdPORTAL. 2024 Sep 12;20:11443. doi: 10.15766/mep_2374-8265.11443 (PMC11390879; doi:10.15766/mep_2374-8265.11443)
Supplement: Supplementary file 1 — Impact of Racism on Health Module.pptxPre- & Posttest.docxFacilitator Guide.docx [file mep_2374-8265.11443-s001.zip › C. Facilitator Guide.docx]

Impact of Racism Workshop Facilitator Guide

This teaching activity should take approximately 1 hour to complete. Use this facilitator guide to prepare for facilitation as it can also be used for a script. You can also refer to this guide while facilitating the module. Areas in red are related to the pretest and posttest so make sure to emphasize these points while presenting.

**Recommended Pre-Reading for Moderator:**

IHI White Paper – Achieving Health Equity

(Wyatt R, Laderman M, Botwinick L, Mate K, Whittington J. *Achieving Health Equity: A Guide for Health Care Organizations*. IHI White Paper. Cambridge, Massachusetts: Institute for Healthcare Improvement; 2016. (Available at ihi.org)

SLIDE 1

I. Speaker Introduction

SLIDE 2

1. Before we dive into the topic today, I want to go over a few housekeeping items.
   1. Please note that the views and ideas expressed in this module do not represent those held by the Department of Defense or United States Armed Forces.
   2. The original content developer attended medical school in Boston, which is relevant to several of the references made in this module.
   3. Please be advised that there will be pre-module and post-module assessments to evaluate the effectiveness of this presentation in addressing the intended learning objectives. Participation is voluntary and all recorded responses are anonymous. This information will be used only to evaluate the intended objective.

SLIDE 3: Learning Objectives

SLIDE 4: Introduction

1. Strongly recommend an acknowledgment that talking about racism is incredibly sensitive for many people and that participation is voluntary. This is also a great time for the group to come up with “community agreements” for the workshop – e.g., listening with respect, everyone has the right to share their perspectives, etc.

SLIDE 5: Health Tips

1. Ask the audience to give examples of common good health behaviors we often counsel our patients in outpatient primary care clinics.
2. Then advance slide to show the prescribed list.
3. If the audience is engaged and participating, you may opt to ask: What is the problem of only focusing on this list?
   1. ANSWER: It narrowly focuses on the individual and ignores the context in which their behaviors are occurring. Think about people engaging in these in YOUR city. Not all people have the same ability to follow these instructions.

SLIDE 6: SDOH

- 1. What if practitioners worked to address the barriers patients experience in undertaking these health promoting behaviors?
  2. Healthy People 2030

SLIDE 7 - 12: CASE

7 For the remainder of this talk, we will use a case to illustrate this point. I could use any chronic disease

process relevant to any of your subspecialties to illustrate this point, but will be focusing on one common pediatric condition instead to help illustrate this point.

8: Clinical Vignette

9: Introduction to questions – only have them answer question #1 here, as they will be addressing the others on subsequent slides

1. The answer to the case diagnosis is intended to be easy.
2. Please emphasize here that the goal is to go beyond diagnosis of the clinical issue, to diagnosis of which social determinants may affect your management of this patient

10: Unmodifiable factors – have participants list some items before you advance to the provided examples 11: Modifiable factors – have participants list some items before you advance to the provided examples 12: Barriers to modifying modifiable risk factors are SDOH we can alter!

SLIDE 13: Racism affects all determinants

SLIDE 14: Define racism

1. Example definition is provided on the slide, but consider adding in the more detailed definition below:
   1. Racism is a system of structuring opportunity and assigning value based on phenotype - “race” that
      1. Unfairly disadvantages some individuals and communities
      2. Unfairly advantages other individuals and communities

SLIDE 15: Levels of racism

1. You can briefly touch on micro level racism, but today we will be focusing on institutional racism which contributes to structural racism
   1. Institutional = refers specifically to the ways in which policies and practices of organizations or parts of systems (schools, courts, transportation authorities, etc.) create different outcomes for different racial groups
   2. Structural = The complex system by which racism is developed, maintained, and protected – all of the aforementioned types of racism
      1. Remember that ‘protection’ of racism, may be less about protecting the disadvantages that one group faces, and more about protecting the advantages that another group receives as a result of racism

SLIDE 16: Structural Racism example

1. I mentioned that structural racism refers to a complex system
2. I’d like to break that system apart to illustrate how structural racism can affect health outcomes for an asthmatic patient like ours
   1. I will do that by describing redlining
3. REDLINING- Data from NPR article in 2017 - A 'Forgotten history' of how the U.S. government segregated America
   1. “The term "redlining" comes from the development by the New Deal, by the federal government of maps of every metropolitan area in the country.
   2. And those maps were color-coded by first the Home Owners Loan Corp and then the Federal Housing Administration and then adopted by the Veterans Administration, and these color codes were designed to indicate where it was safe to insure mortgages.
   3. And anywhere where African-Americans lived, anywhere where African-Americans lived nearby were colored red to indicate to appraisers that these neighborhoods were too risky to insure mortgages
   4. This map is an image of the red lining
      1. Red areas were those neighborhoods that weren’t safe to ensure – Roxbury and N. Dorchester
   5. This redlining led to divestment from black neighborhoods.

SLIDE 17: Asthma ED visits

1. Point out that asthma ED visits share a similar distribution to redlined areas
2. And in the subsequent slides we will explain how that came to be

SLIDE 18-19: Environmental exposure

1. The first example of how structural racism affected social determinants of health was its result in unequal environmental exposures
   1. The mechanism: As supermarkets and businesses left these areas, there was more room for waste deposition
      1. Junkyards are a major source of pollution in cities
      2. Junkyard locations correspond to the areas where asthma hospitalizations are higher
   2. The result: In communities of color there are an average of 192,000 pounds of chemical pollution per square mile versus 19,000 in white communities
   3. We know that air pollutant exposure is a risk factor/trigger for asthma

SLIDE 20: Lack of green space

1. Also related to environmental exposure
   1. Children who spend more time indoors are exposed to more indoor allergens such as dust and mold. Therefore, parks and green space are important health promoting resources in communities – to reduce air pollutants and provide opportunities for kids to get outside.
2. The mechanism: People didn’t want to invest $$$ into neighborhoods that were uninsured and also couldn’t afford maintenance of green space
   1. The results: neighborhoods with higher asthma rates also have less green space than other neighborhoods

SLIDE 21-22: Safe and affordable housing

1. These homes weren’t insured, therefore no routine maintenance
2. Poor housing stock  more indoor allergens such as mold
   1. Also lead to higher lead levels
   2. When we look at the geographic distribution of elevated blood lead levels, we see a relationship with the neighborhoods that have the highest rates of asthma.

SLIDE 23-24: SES

1. Federal Poverty Line Density is in center of the city, same areas with less green space, more junkyards and higher rates of asthma

SLIDE 25-26: Access to health services

1. Extrapolating the fact that asthma exacerbation hospitalizations can be considered a failure of primary care in the case of asthma care
2. If you look at hospitalizations for asthma exacerbation, much higher for black and latinx patients compared to all other races

SLIDE 27: Putting It Together

- - One’s race affects the places where they live, their job opportunities, their environmental exposures, access to health services (COVID testing sites have been located in richer white neighborhoods), safe and affordable housing and exposure to violence. All of which contribute to health outcomes for a myriad of diseases. Asthma is only one example. Other similarly well-established racial disparities exist in chronic disease hospitalizations and low birth weight to mention just a few.

SLIDE 28-29: MHS Outcomes

- - Ask if the audience has seen racial/ethnic disparities in children with asthma in the MHS.
  - Advance the slide – this is a JAMA Pediatrics study from 2010 looking at disparities in prevalence, treatment, and outcomes of asthma in a group of children that receive care within the MHS.
    - As you can see, there are disparities in the prevalence of asthma diagnosis, settings of care, and treatment for Black and Hispanic Children.
  - Slide 30 is an excerpt from the paper that discussed disparities in sponsor rank in accordance with the health disparities that were seen in the study and also a suggestion that the black children in the study had worse control over their asthma and were not using medications appropriately.

SLIDE 30-31: Back to the case

1. Have participants break into smaller groups of 4-6 to address these questions.
   1. Then come back as a larger group and debrief. For time purposes, suggest 1-2 volunteers per question.
2. These questions are meant to initiate a discussion about the importance of framing a disease in the context of the patient’s lived experience.
   1. Patients often feel guilt for their physical ill-health, and to the extent that we as providers can free them of that guilt, it may be argued we can build a stronger therapeutic relationship.
   2. There are numerous examples where systems-thinking physicians may improve health of their patient population outside the acute care setting.
      1. General Example: More active roles in policy making at the governmental level, for example, explaining the role of reliable public transportation to lawmakers and its potential benefit to the outcomes of dialysis patients making their appointments.
      2. Military-Specific Structures to Emphasize in Debriefing:
         - Makeup of the Enlisted vs Officer Corps
           - There are more ethnic and racial minorities in the enlisted corps vs the officer corps across ALL military branches in the United States. Becoming an officer requires a degree at the minimum which can exacerbate disparities according to rank.
         - Education Funding in Home Communities & Health Literacy
           - Individuals that join the military as enlisted servicemembers generally join for upward social mobility and opportunities. If coming from an area with limited educational opportunities because of limited funding in their community (e.g, inner city urban areas or rural area), these individuals may become parents with low health literacy which can impact the ways in which their children access the healthcare system and use medications (e.g, use of primary care vs emergency room; appropriate use of rescue vs controller medications, etc.)
         - Current Housing Policies & Environmental Exposures
           - Environmental exposures (mold and rodent infestations) in on-base privatized housing (which is a common issue!) which disproportionately affects junior enlisted servicemembers and their families.
           - Environmental exposures in off-base housing in areas that require higher per-diem which disproportionately affects junior enlisted servicemembers and their families.
           - This is an excellent opportunity for advocacy that one can get involved in as a military physician.

SLIDE 32-33: SUMMARY

- - Review the points listed, but don’t forget to ask participants what lessons THEY learned that may not be included in our summary.
