# Supplementary material for: The Impact of Racism on Health: A Health Equity Training on Structural Racism for Military Residents and Fellows
Source: MedEdPORTAL. 2024 Sep 12;20:11443. doi: 10.15766/mep_2374-8265.11443 (PMC11390879; doi:10.15766/mep_2374-8265.11443)
Supplement: Supplementary file 1 — Impact of Racism on Health Module.pptxPre- & Posttest.docxFacilitator Guide.docx [file mep_2374-8265.11443-s001.zip › B. Pre- & Posttest.docx]

**Impact of Racism on Health**

**Pretest and Posttest**

**This knowledge assessment is to be used prior to presenting didactics material (usually after disclosure slide) and again at the end of the teaching activity. It should take approximately 5 minutes to administer.**

1. Which of the following is correct about the process of redlining?

# Areas adjacent to African American communities were colored red to indicate to appraisers that these neighborhoods were too risky to insure mortgages

- 1. Areas adjacent to White communities were colored red to indicate high value properties.
  2. Areas adjacent to high rates of violence and crime were colored red to discourage people from living there.
  3. Areas adjacent to the best schools that were colored red to encourage investments in these neighborhoods.

1. Which of the following resulted from redlining? Select all that apply.

# Poor housing stock associated with higher blood lead levels in communities of color.

- 1. Improved access to education in communities where redlining occurred.

# Poor air quality found more commonly in communities of color.

- 1. Improved access to appropriate insurance coverage in areas where redlining occurred.

1. Which of the following is a modifiable risk factor for asthma? Select all that apply
   1. Age
   2. Race

# Exercise

- 1. Family history
  2. **Exposure to extreme temperatures**
  3. **Exposure to pests**
